# Supplementary material for: Do hospitals influence geographic variation in admission for preventable hospitalisation? A data linkage study in New South Wales, Australia
Source: BMJ Open. 2019 Feb 22;9(2):e027639. doi: 10.1136/bmjopen-2018-027639 (PMC6398792; doi:10.1136/bmjopen-2018-027639)
Supplement: Supplementary data [file bmjopen-2018-027639supp001.pdf]

Online appendices

Appendix 1:

ICD-10-AM codes for identifying hospital outcomes.

Category	ICD-10-AM diagnosis and procedure codes
**Preventable hospitalisations**	
Angina	I20, I24.0, I24.8, I24.9 as principal diagnosis only, exclude cases with procedure codes not in blocks [1820] to [2016]
Asthma	J45, J46 as principal diagnosis only
Chronic obstructive pulmonary disease (COPD)	J20, J41, J42, J43, J44, J47 as principal diagnosis only, J20 only with additional diagnoses of J41, J42, J43,J44, J47
Congestive cardiac failure	I50, I11.0, J81 as principal diagnosis only, exclude cases with the following procedure codes: 33172-00, 35304-00, 35305-00, 35310-02, 35310-00, 38281-11, 38281-07, 38278-01, 38278-00, 38281-02, 38281-01, 38281-00, 38256-00, 38278-03, 38284-00, 38284-02, 38521-09, 38270-01, 38456-19, 38456-15, 38456-12, 38456-11, 38456-10, 38456-07, 38456-01, 38470-00, 38475-00, 38480-02, 38480-01, 38480-00, 38488-06, 38488-04, 38489-04, 38488-02, 38489-03, 38487-00, 38489-02, 38488-00, 38489-00, 38490-00, 38493-00, 38497-04, 38497-03, 38497-02, 38497-01, 38497-00, 38500-00, 38503-00, 38505-00, 38521-04, 38606-00, 38612-00, 38615-00, 38653-00, 38700-02, 38700-00, 38739-00, 38742-02, 38742-00, 38745-00, 38751-02, 38751-00, 38757-02, 38757-01, 38757-00, 90204-00, 90205-00, 90219-00, 90224-00, 90214-00, 90214-02.
Diabetes complications	E10–E14.9 as principal diagnoses, and E10–E14.9 as additional diagnoses where the principal diagnosis was: hypersmolarity (E87.0), acidosis (E87.2), transient ischaemic attack (G45), nerve disorders and neuropathies (G50–G64), cataracts and lens disorders (H25–H28), retinal disorders (H30–H36), glaucoma (H40–H42), myocardial infarction (I21–I22), other coronary heart diseases (I20, I23–I25), heart failure (I50), stroke and sequelae (I60–I64, I69.0–I69.4), peripheral vascular disease (I70–I74), gingivitis and periodontal disease (K05), kidney diseases including end-stage renal disease (N00–N29), and renal dialysis (Z49)
Hypertension	I10, I11.9 as principal diagnosis only, exclude cases with procedure codes according to the list of procedures excluded from the Congestive cardiac failure category above.
Iron deficiency anaemia	D50.1, D50.8, D50.9 as principal diagnosis only.
Nutritional deficiencies	E40, E41, E42, E43, E55.0, E64.3 as principal diagnosis only.
Rheumatic heart disease	I00 to I09 as principal diagnosis only. (Note: includes acute rheumatic fever)
Appendicitis with generalised peritonitis	K35.0 in any diagnosis field
Cellulitis	L03, L04, L08, L88, L98.0, L98.3 as principal diagnosis only, exclude cases with any procedure except those in blocks 1820 to 2016 or if procedure is 30216-02, 30676-00, 30223-02, 30064-00, 34527-01, 34527-00, 90661-00 and this is the only listed procedure
Convulsions and epilepsy	G40, G41, O15, R56 as principal diagnosis only
Dehydration and gastroenteritis	A09.9, E86, K52.2, K52.8, K52.9 as principal diagnosis only.
Dental conditions	K02, K03, K04, K05, K06, K08, K09.8, K09.9, K12, K13 as principal diagnosis only.
Ear, nose and throat infections	H66, H67, J02, J03, J06, J31.2 as principal diagnosis only.
Gangrene	R02 in any diagnosis field
Pelvic inflammatory disease	N70, N73, N74 as principal diagnosis only.
Perforated/bleeding ulcer	K25.0, K25.1, K25.2, K25.4, K25.5, K25.6, K26.0, K26.1, K26.2, K26.4, K26.5, K26.6, K27.0, K27.1, K27.2, K27.4, K27.5, K27.6, K28.0, K28.1, K28.2, K28.4, K28.5, K28.6 as principal diagnosis only.
Pyelonephritis	N10, N11, N12, N13.6, N39.0 as principal diagnosis only.
Influenza and pneumonia	J10, J11, J13, J14, J15.3, J15.4, J15.7, J15.9, J16.8, J18.1, J18.8 in any diagnosis field, excludes cases with additional diagnosis of D57 (sickle-cell disorders) and people under 2 months
Other vaccine-preventable conditions	A35, A36, A37, A80, B05, B06, B16.1, B16.9, B18.0, B18.1, B26, G00.0, M01.4 in any diagnosis field
Acute myocardial infarction (AMI)	I21
Hip fracture	S72.0, S72.1, S72.2

Appendix 2:

Hospital categories, corresponding peer groups from the *NSW Health Services Comparison Data Book 2008/2009*, and number all cause admissions during follow-up.

Hospital Peer Group	Description	Admissions
**Principal**		
A1a Principal Referral Group A	Acute hospitals, treating 25,000 or more acute casemix weighted separations per annum, with an average cost weight greater than 1 and having more than 1 specialty service.	76,193
A1b Principal Referral Group B	Acute hospitals, treating 25,000 or more acute casemix weighted separations per annum, with an average cost weight greater than 1 and 1 or fewer specialty services.	28,424
A2 Paediatric Specialist	Establishments where the primary role is to provide specialist acute care services for children.	-
A3 Ungrouped Acute	Establishments whose primary role is the provision of acute services of a specialised nature for which there is insufficient peers to form additional peer groups.	6,284
**Major metropolitan**		
B1 Major Metropolitan:	Acute hospitals, treating 10,000 or more acute casemix weighted separations per annum, but having less than 25,000 acute casemix weighted separations or an average casemix weight of less than 1.	30,512
**Major non-metropolitan**		
B2 Major Non-Metropolitan:	Acute hospitals treating 10,000 or more acute casemix weighted separations per annum that are located in rural areas providing acute specialist and referral services for a catchment population from a large geographical area.	68,460
**District**		
C1 District Group 1	Acute hospitals, treating 5,000 or more, but less than 10,000 acute casemix weighted separations per annum.	27,671
C2 District Group 2	Acute hospitals, treating 2,000 or more, but less than 5,000 acute casemix weighted separations per annum, plus acute hospitals treating less than 2,000 acute casemix weighted separations per annum but with more than 2,000 separations per annum.	29,470
**Community**		
D1a Community Acute with Surgery	Acute hospitals, treating less than 2,000 acute casemix weighted separations per annum, and less than 2,000 acute separations per annum, with less than 40% nonacute and outlier bed days of total bed days and greater than 2% of their acute weighted separations being surgical.	6,352
D1b Community Acute without Surgery	Acute hospitals, treating less than 2,000 acute casemix weighted separations per annum, and less than 2,000 acute separations per annum, with less than 40% nonacute and outlier bed days of total bed days, and less than 2% of their acute weighted separations being surgical.	3,620
D2 Community Non-Acute	Non-acute hospitals, treating less than 2,000 acute casemix weighted separations per annum, and less than 2,000 acute separations per annum, with more than 40% nonacute and outlier bed days of total bed days.	3,061
**Multi-purpose**		
F1 Psychiatric	Establishments devoted primarily to the treatment and care of inpatients with psychiatric, mental or behavioural disorders. Centres of non-acute treatment of drug dependence, developmental and intellectual disability are not included here. This group also excludes institutions mainly providing living quarters or day care.	195
F2 Nursing Homes	Establishments which provide long-term care involving regular base nursing care to chronically ill, frail, disabled or convalescent persons or senile inpatients. They must be approved by the Commonwealth Department of Health and Family Services and /or licensed by the State, or controlled by government departments.	184
F3 Multi-Purpose Services	Multi-Purpose Services (MPSs) which provide integrated acute health, nursing home, hostel, community health and aged care services under one organisational structure, as agreed between the Commonwealth and State Governments. MPSs provide a range of services which are negotiated with the community, the service providers and the relevant Departments.	4,493
F4 Sub Acute	Establishments that primarily provide sub-acute services, but are not specialist palliative care or specialist rehabilitation establishments.	2,588
F5 Palliative Care	Establishments with a specific function of providing palliative care to terminally ill patients.	86
F6 Rehabilitation	Establishments with a primary role in providing services to persons with an impairment, disability or handicap where the primary goal is improvement in functional status.	34
F7 Mothercraft	Establishments where the primary role is to help mothers acquire mothercraft skills in an inpatient setting.	5
F8 Ungrouped Non-Acute	Establishments whose primary role is the provision of non-acute services, but for which there are insufficient peers to form an addition peer group. Limited comparisons can be made within this peer group and with other non-acute facilities.	328

Appendix 3:

Incidence rate ratio (IRR) of patient-, area- and hospital-level factors from cross-classified multiple membership multilevel Poisson models on preventable hospitalisations and emergency admissions for acute myocardial infarction (AMI) and hip fracture

	Preventable hospitalisation		AMI (emergency)		Hip fracture (emergency)	
	IRR	(95% CIs)	IRR	(95% CIs)	IRR	(95% CIs)
**Patient-level factors**						
Age						
45-54 years	1.00	(ref)	1.00	(ref)	1.00	(ref)
55-64 years	1.23	(1.18 - 1.29)	1.71	(1.49 - 1.97)	2.25	(1.52 - 3.33)
65-74 years	1.72	(1.64 - 1.81)	2.39	(2.05 - 2.80)	7.12	(4.89 - 10.4)
75-84 years	2.57	(2.44 - 2.70)	4.23	(3.61 - 4.96)	24.7	(17.1 - 35.7)
85 years and over	3.40	(3.20 - 3.61)	7.64	(6.35 - 9.18)	49.2	(33.7 - 71.6)
Sex						
Males	1.00	(ref)	1.00	(ref)	1.00	(ref)
Females	0.72	(0.70 - 0.74)	0.42	(0.39 - 0.46)	1.36	(1.21 - 1.53)
Highest level of education						
Did not complete high school	1.00	(ref)	1.00	(ref)	1.00	(ref)
High school or equivalent	0.93	(0.91 - 0.96)	0.92	(0.85 - 0.99)	1.02	(0.91 - 1.15)
University or higher	0.86	(0.83 - 0.90)	0.78	(0.69 - 0.88)	1.16	(0.98 - 1.38)
Unknown / missing	1.13	(1.07 - 1.20)	0.95	(0.77 - 1.16)	0.90	(0.68 - 1.18)
Marital status						
Single	1.00	(ref)	1.00	(ref)	1.00	(ref)
Married / partnered	1.19	(1.13 - 1.25)	1.09	(0.93 - 1.28)	1.31	(1.05 - 1.63)
Widowed / divorced / separated	1.17	(1.13 - 1.20)	1.19	(1.10 - 1.30)	1.14	(1.01 - 1.28)
Unknown / missing	1.22	(1.08 - 1.37)	0.79	(0.50 - 1.25)	0.57	(0.25 - 1.29)
Annual household income						
<$10,000	1.00	(ref)	1.00	(ref)	1.00	(ref)
$10,000 - $29,999	0.89	(0.86 - 0.92)	0.87	(0.76 - 0.99)	1.04	(0.87 - 1.25)
$30,000 - $49,000	0.80	(0.76 - 0.84)	0.89	(0.76 - 1.04)	0.80	(0.62 - 1.02)
$50,000 - $69,999	0.75	(0.70 - 0.80)	0.85	(0.70 - 1.03)	0.88	(0.63 - 1.21)
$70,000 or more	0.65	(0.61 - 0.69)	0.75	(0.62 - 0.90)	0.85	(0.63 - 1.16)
Not specified	0.94	(0.90 - 0.98)	1.04	(0.90 - 1.20)	1.05	(0.86 - 1.28)
Unknown / missing	1.13	(1.07 - 1.18)	1.14	(0.97 - 1.34)	1.15	(0.93 - 1.43)
Employment status						
Not working	1.00	(ref)	1.00	(ref)	1.00	(ref)
Part time	0.82	(0.79 - 0.86)	0.90	(0.78 - 1.04)	0.63	(0.47 - 0.84)
Full time	0.84	(0.80 - 0.88)	1.16	(1.01 - 1.33)	0.90	(0.67 - 1.19)
Missing / unknown	0.93	(0.85 - 1.01)	1.03	(0.78 - 1.38)	0.91	(0.64 - 1.28)
Language spoken at home						
English	1.00	(ref)	1.00	(ref)	1.00	(ref)
Other	0.92	(0.88 - 0.96)	0.99	(0.87 - 1.12)	1.04	(0.88 - 1.23)
Health insurance status						
Private with extras	1.00	(ref)	1.00	(ref)	1.00	(ref)
Private, no extras	1.03	(0.99 - 1.08)	1.05	(0.93 - 1.18)	0.93	(0.79 - 1.09)
Department of Veterans Affairs	1.28	(1.21 - 1.36)	1.08	(0.91 - 1.28)	1.11	(0.91 - 1.35)
Health Care Card	1.60	(1.54 - 1.65)	1.42	(1.29 - 1.57)	0.94	(0.82 - 1.08)

	Preventable hospitalisation		AMI (emergency)		Hip fracture (emergency)	
	IRR	(95% CIs)	IRR	(95% CIs)	IRR	(95% CIs)
None	1.50	(1.44 - 1.55)	1.26	(1.13 - 1.41)	0.93	(0.78 - 1.10)
Number of people can depend on						
0 people	1.00	(ref)	1.00	(ref)	1.00	(ref)
1-4 people	1.09	(1.04 - 1.14)	0.92	(0.80 - 1.06)	1.20	(0.96 - 1.49)
5-10 people	1.07	(1.02 - 1.12)	0.92	(0.80 - 1.07)	1.18	(0.94 - 1.47)
11 or more people	1.20	(1.14 - 1.27)	0.89	(0.75 - 1.06)	1.15	(0.89 - 1.50)
Unknown / missing	1.16	(1.10 - 1.24)	0.99	(0.82 - 1.20)	1.35	(1.04 - 1.77)
Positive health seeking behaviours [a]						
0 health behaviours	0.96	(0.86 - 1.07)	1.02	(0.72 - 1.43)	1.31	(0.77 - 2.23)
1 health behaviour	1.00	(ref)	1.00	(ref)	1.00	(ref)
2 health behaviours	0.89	(0.86 - 0.93)	0.89	(0.80 - 1.00)	0.76	(0.65 - 0.89)
3 health behaviours	0.78	(0.75 - 0.81)	0.76	(0.68 - 0.85)	0.65	(0.55 - 0.77)
4 health behaviours	0.76	(0.72 - 0.80)	0.75	(0.64 - 0.88)	0.55	(0.43 - 0.70)
BMI						
Underweight	1.13	(1.08 - 1.17)	1.01	(0.88 - 1.15)	1.10	(0.96 - 1.27)
Healthy weight	1.00	(ref)	1.00	(ref)	1.00	(ref)
Overweight	0.94	(0.91 - 0.97)	0.95	(0.87 - 1.03)	0.57	(0.50 - 0.64)
Obese	1.00	(0.96 - 1.03)	1.00	(0.90 - 1.10)	0.35	(0.29 - 0.42)
Unknown / missing	1.20	(1.10 - 1.32)	1.10	(0.80 - 1.50)	0.69	(0.41 - 1.16)
Self-rated health						
Excellent	1.00	(ref)	1.00	(ref)	1.00	(ref)
Very good	1.22	(1.13 - 1.30)	1.21	(1.03 - 1.43)	0.86	(0.67 - 1.10)
Good	1.60	(1.50 - 1.71)	1.32	(1.12 - 1.55)	1.04	(0.81 - 1.33)
Fair	2.58	(2.40 - 2.76)	1.59	(1.33 - 1.90)	1.18	(0.91 - 1.54)
Poor	4.10	(3.79 - 4.43)	1.84	(1.47 - 2.32)	1.66	(1.20 - 2.29)
Unknown / missing	2.27	(2.09 - 2.46)	1.37	(1.09 - 1.72)	1.04	(0.77 - 1.42)
Multi-morbid conditions [b]						
No conditions	1.00	(ref)	1.00	(ref)	1.00	(ref)
1 condition	1.35	(1.31 - 1.40)	1.20	(1.09 - 1.32)	1.00	(0.87 - 1.14)
2 conditions	1.98	(1.91 - 2.05)	1.45	(1.30 - 1.62)	0.90	(0.77 - 1.05)
3 or more conditions	2.71	(2.60 - 2.82)	2.26	(2.01 - 2.55)	1.06	(0.90 - 1.26)
Functional limitations [c]						
No limitation	1.00	(ref)	1.00	(ref)	1.00	(ref)
Minor limitations	1.04	(0.97 - 1.10)	0.97	(0.84 - 1.14)	0.83	(0.60 - 1.16)
Mild limitation	1.25	(1.18 - 1.32)	1.04	(0.91 - 1.19)	1.13	(0.86 - 1.48)
Moderate limitation	1.56	(1.48 - 1.64)	1.17	(1.03 - 1.34)	1.86	(1.45 - 2.38)
Severe limitation	2.36	(2.24 - 2.48)	1.49	(1.30 - 1.71)	2.83	(2.21 - 3.63)
Unknown / missing	1.71	(1.61 - 1.80)	1.24	(1.07 - 1.44)	1.75	(1.36 - 2.26)
Psychological distress [d]						
Low distress	1.00	(ref)	1.00	(ref)	1.00	(ref)
Moderate distress	1.03	(0.99 - 1.06)	1.01	(0.91 - 1.12)	1.03	(0.88 - 1.20)
High distress	0.99	(0.95 - 1.03)	1.08	(0.92 - 1.26)	1.10	(0.87 - 1.39)
Very high distress	0.99	(0.94 - 1.06)	0.88	(0.69 - 1.12)	1.18	(0.83 - 1.66)
Unknown / missing	1.09	(1.03 - 1.15)	1.31	(1.12 - 1.54)	1.16	(0.95 - 1.42)
**Area-level factors**						
Remoteness of residence						

	Preventable hospitalisation		AMI (emergency)		Hip fracture (emergency)	
	IRR	(95% CIs)	IRR	(95% CIs)	IRR	(95% CIs)
Major city	1.00	(ref)	1.00	(ref)	1.00	(ref)
Inner regional	0.95	(0.88 - 1.02)	1.05	(0.91 - 1.22)	1.13	(0.95 - 1.33)
Outer regional	1.01	(0.88 - 1.15)	1.08	(0.87 - 1.34)	1.06	(0.81 - 1.38)
Remote / very remote	1.24	(0.97 - 1.58)	0.95	(0.61 - 1.48)	0.84	(0.44 - 1.62)
Full-time workload equivalent GPs						
Quintile 1 (2.64-6.90 GPs)	1.00	(ref)	1.00	(ref)	1.00	(ref)
Quintile 2 (6.91-7.60 GPs)	0.89	(0.78 - 1.02)	0.88	(0.73 - 1.07)	1.01	(0.80 - 1.29)
Quintile 3 (7.63-8.64 GPs)	0.95	(0.84 - 1.08)	0.89	(0.74 - 1.08)	1.10	(0.87 - 1.39)
Quintile 4 (8.65-9.94 GPs)	0.92	(0.79 - 1.06)	0.88	(0.72 - 1.07)	1.04	(0.82 - 1.32)
Quintile 5 (9.95-13.3 GPs)	1.02	(0.87 - 1.19)	0.91	(0.74 - 1.12)	0.96	(0.74 - 1.23)
**Hospital-level factors**						
Hospital category						
Principal	1.00	(ref)	1.00	(ref)	1.00	(ref)
Major metropolitan	0.99	(0.95 - 1.03)	1.02	(0.99 - 1.05)	1.02	(0.99 - 1.05)
Major non-metropolitan	1.01	(0.97 - 1.04)	1.04	(1.02 - 1.07)	0.99	(0.96 - 1.02)
District	1.02	(0.99 - 1.06)	1.00	(0.97 - 1.03)	0.99	(0.96 - 1.02)
Community	1.06	(1.02 - 1.10)	0.97	(0.93 - 1.01)	0.96	(0.91 - 1.01)
Multipurpose	1.05	(1.01 - 1.09)	0.93	(0.88 - 0.99)	1.02	(0.94 - 1.09)
**Random effects**						
Residual random effect (SE)						
Hospital-level	0.276	(0.056)	0.010	(0.013)	0.024	(0.022)
Area-level	0.061	(0.011)	0.050	(0.015)	0.013	(0.011)

[a] Healthy behaviours, of non-smoking status, safe level of alcohol consumption (<14 drinks per week), at least 2.5 hours of intensity-weighted physical activity per week, and meeting dietary guidelines for daily fruit (2 serves) and vegetable (5 serves) consumption

[b] Of self-reported heart disease, high blood pressure, stroke, diabetes, blood clot, asthma, Parkinson's disease, and any cancer except skin cancer.

[c] Measured using the Medical Outcome Study physical functioning scale.

[d] Measured using the K10 scale.
