# Supplementary material for: Do hospitals influence geographic variation in admission for preventable hospitalisation? A data linkage study in New South Wales, Australia
Source: BMJ Open. 2019 Feb 22;9(2):e027639. doi: 10.1136/bmjopen-2018-027639 (PMC6398792; doi:10.1136/bmjopen-2018-027639)
Supplement: Author's manuscript [file bmjopen-2018-027639.draft_revisions.pdf]

BMJ Open is committed to open peer review. As part of this commitment we make the peer review history of every article we publish publicly available.

When an article is published we post the peer reviewers' comments and the authors' responses online. We also post the versions of the paper that were used during peer review. These are the versions that the peer review comments apply to.

The versions of the paper that follow are the versions that were submitted during the peer review process. They are not the versions of record or the final published versions. They should not be cited or distributed as the published version of this manuscript.

BMJ Open is an open access journal and the full, final, typeset and author-corrected version of record of the manuscript is available on our site with no access controls, subscription charges or pay-per-view fees (http://bmjopen.bmj.com).

If you have any questions on BMJ Open's open peer review process please email info.bmjopen@bmj.com

BMJ Open

# Exploring hospital variation in preventable hospitalisation in Australia

| | |
|---|---|
| Journal: | *BMJ Open* |
| Manuscript ID | bmjopen-2018-027639 |
| Article Type: | Research |
| Date Submitted by the Author: | 01-Nov-2018 |
| Complete List of Authors: | Falster, Michael; University of New South Wales, Centre for Big Data Research in Health<br>Leyland, Alastair; MRC/CSO Social and Public Health Sciences Unit, University of Glasgow,<br>Jorm, Louisa; University of New South Wales, Centre for Big Data Research in Health |
| Keywords: | PRIMARY CARE, Preventable hospitalisations, Ambulatory care sensitive conditions, Multilevel modelling |
| | |

SCHOLARONE™
Manuscripts

# Title

Exploring hospital variation in preventable hospitalisation in Australia

# Authors

Michael O Falster, PhD[1]; Alastair H Leyland, PhD[2]; Louisa R Jorm, PhD[1];
[1] Centre for Big Data Research in Health, UNSW Sydney, Australia
[2] MRC/CSO Social and Public Health Sciences Unit, University of Glasgow, Glasgow UK

# Corresponding author

Dr Michael O. Falster
Centre for Big Data Research in Health, UNSW Sydney
Level 1, AGSM Building (G27), UNSW
Sydney NSW 2052, Australia
+61 (2) 9385 0698
m.falster@unsw.edu.au

For peer review only

# Abstract

**Objective**: Preventable hospitalisations are used internationally as a performance indicator for primary care, but the influence of other health system factors remains poorly understood. This study investigated between-hospital variation in rates of preventable hospitalisation.

**Setting**: Linked health survey and hospital admissions data for a cohort study of 266,826 people aged over 45 years in the state of New South Wales, Australia.

**Method**: Between-hospital variation in preventable hospitalisation was quantified using cross-classified multiple-membership multilevel Poisson models, adjusted for personal sociodemographic, health and area-level contextual characteristics. Variation was also explored for two conditions unlikely to be influenced by discretionary admission practice: emergency admissions for acute myocardial infarction (AMI) and hip fracture.

**Results**: We found significant between-hospital variation in adjusted rates of preventable hospitalisation, with hospitals varying on average 26% from the state mean. Patients served more by community and multipurpose facilities (smaller facilities primarily in rural areas) had higher rates of preventable hospitalisation. Community hospitals had the greatest between-hospital variation, and included the facilities with the highest rates of preventable hospitalisation. There was comparatively little between-hospital variation in rates of admission for AMI and hip fracture.

**Conclusions**: Geographic variation in preventable hospitalisation is determined in part by hospitals, reflecting different roles played by community and multipurpose facilities, compared with major and principal referral hospitals, within the community. The indicator cannot be interpreted simply as a performance measure for primary care.


## Strengths and limitations of the study:

- The use of multilevel modelling with detailed patient survey data make this the first study on preventable hospitalisations to have appropriately modelled each of patient-, area- and hospital-level effects.
- There remains unexplained between-hospital variation, and the impact of more complex models of care has yet to be explored
- The study population is not representative of the Australian population, being an older cohort (age 45 and over) with a low response rate



For peer review only - http://bmjopen.bmj.com/site/about/guidelines.xhtml

For peer review only

# Introduction

Preventable hospitalisations are an intuitive, yet contentious, performance indicator for primary care. Also known as hospital admissions for ambulatory care sensitive conditions, rates of preventable hospitalisations are used in Australia[1,2] and internationally as a measure of hospital use that could potentially be prevented through timely and effective access to primary care. These admissions are estimated to cost over $30 billion dollars annually in the US,[3] presenting significant potential cost savings to the healthcare system. However, rates of preventable hospitalisation in Australia have not declined, despite accounting for 6% of all hospitalisations and being a national performance indicator for over 10 years.[4]

Health system performance measures should be underpinned by strong evidence that improvements will lead to improvements in health outcomes,[5] and the utility of preventable hospitalisations as a performance measure has been challenged accordingly.[6] Initially developed in the US where large variations in income, workforce and health insurance coverage result in stark disparities in access to primary care,[7,8] the subsequent adoption of the indicator in various international settings has produced a mixed evidence base, particularly in countries with a universal health care system such as Australia[9], Canada[10,11] and the UK.[6] The utility of the indicator is likely to differ according to the characteristics of the patient population, and the barriers and facilitators to accessing care in the health system.

One health system factor which remains poorly understood is the role of hospitals. Differences in a hospital's propensity to admit patients can arise from physician preferences[12] and in-hospital capacity.[7,13,14] Anecdotal reports from the UK suggest that hospitals play a direct role in choosing to admit patients for observation, such as in regional areas where long travel times and limited clinical support can lead to more cautious admission thresholds.[15] Australia has a vast geography, and in remote areas hospitals and emergency departments may be used as a substitute for GP care.[16] However evidence on hospitals' influence on preventable hospitalisations is limited: higher rates have been reported in UK hospitals that convert more emergency department presentations into admissions,[17] and in areas in the US with more hospital beds per capita[18] – although the latter finding has been inconsistent.[19,20]

A better understanding of the role of hospitals would improve our understanding of the limitations of preventable hospitalisations as an indicator of primary care. We sought to quantify between-hospital variation in preventable hospitalisation in New South Wales (NSW), Australia, and assess if this variation differs between categories of hospital facilities.

# Methods

### *Study population*

This observational study included participants in The Sax Institute's 45 and Up Study, a prospective cohort of 267,014 residents of NSW, Australia, aged 45 and over.[21] Eligible participants were randomly selected between 2006-2009 through the Department of Human Services enrolment database. At study entry participants completed a detailed questionnaire containing information on their health and sociodemographic characteristics, and provided informed consent for long-term follow-up, including linkage with administrative health data sets, and use of their data for research purposes.



For peer review only - http://bmjopen.bmj.com/site/about/guidelines.xhtml

For each participant, linked data on hospital admissions (between 2000-2011) and deaths (between 2006-2011) were obtained from the NSW Admitted Patient Data Collection and the NSW Registry of Births Deaths and Marriages mortality data file, respectively. Data linkage was performed probabilistically by the NSW Centre for Health Record Linkage (http://www.cherel.org.au/). Participants were excluded if they had an unknown age, area of residence, or inconsistent records suggesting incorrect linkage (e.g. death before date of study entry).

Ethics approval for the 45 and Up Study was given by the University of New South Wales Human Research Ethics Committee, and ethics approval for this study was given by the NSW Population and Health Services Research Ethics Committee and the University of Western Sydney Research Ethics Committee. All analyses were carried out in accordance with these approvals.

### *Hospitalisations, outcomes and exposures*

Hospital outcomes were identified using the linked hospital admissions data, from the time of participants' entry into the study (between 2006-2009) until death or the end of linked data (31/12/2011), whichever came first. Hospital admissions were restricted to public hospitals only. Transfers and changes in type of care (e.g. from acute to palliative) within a hospital were considered a continuation of the same episode of care.

Preventable hospitalisations were identified according to the 'selected potentially preventable hospitalisations' indicator in the Australian National Healthcare Agreement, a composite measure of hospital admissions for 21 conditions.[22] Two additional outcome measures, for which hospital admission was unlikely to be influenced by discretionary patterns of care, were used for comparison: emergency admissions for acute myocardial infarction (AMI) and hip fracture.[14] Hospital diagnosis and procedure codes used to identify outcomes are in Appendix 1. Sensitivity analyses tested a recently suggested modification to the preventable hospitalisations indicator, categorising preventable hospitalisations as short (<= 2 days length of stay [LOS]) and long (3+ days LOS), on the basis that shorter admissions may be more amenable to primary prevention.[23]

All person-level information was derived from the self-reported survey completed at study entry, including participants' age, sex, education, marital status, annual household income, employment, language spoken at home, health insurance status, level of social support, body mass index, healthy behaviours, multi-morbidity, functional limitation, self-rated health and psychological distress. These variables reflect patients' predisposition and need to use health services, with most previously found to be associated with preventable hospitalisation.[9] All variables were treated as categorical, with missing values as an additional category.[9]

Area-level information was assigned according to the Statistical Local Area (SLA) of patient residence: geographic remoteness used the Accessibility/Remoteness Index of Australia, and the effective supply of full-time workload equivalent general practitioners (GPs), derived from aggregated Medicare claims data.[9,24]

Hospital category was classified according to hospital peer group, a categorisation used for benchmarking and reporting that groups hospitals by the types of services provided.[25] For this analysis, peer groups were collapsed into six broad categories reflecting major differences in the size, role and location of hospitals: principal (>25,000 acute separations per annum), major metropolitan (10-25,000 acute separations per annum), major non-metropolitan (10,000+ acute


For peer review only - http://bmjopen.bmj.com/site/about/guidelines.xhtml

separations per annum, in rural areas), district (2-10,000 acute separations per annum), community (<2,000 acute separations per annum) and multipurpose (smaller facilities providing integrated acute health, nursing home, hostel, community health, aged care and non-specialised sub-acute services) (detailed definitions in Appendix 2).

### *Statistical methods*

Between-hospital variation in admission was analysed using cross-classified multiple membership multilevel Poisson models.[26] All models used number of hospitalisations as the outcome and log of the follow up time as an offset, so as to model 'rates' of admission, and were adjusted for participants' sociodemographic and health characteristics, geographic remoteness and supply of GP services in their area of residence, so the remaining residual variation was that potentially attributable to hospitals.

Multilevel models allow for variation to be partitioned to various 'levels' for analysis, and these models clustered study participants in both their geographic area of residence (SLA) and all potential hospitals of admission. Because a patient could be admitted to any number of hospitals, this clustering was performed using weighted hospital service area networks of all public hospitals servicing the population.[26] Weighting was determined by patterns of patient flow for all-cause admissions at the level of the postal-area.

From these models, hospital-level incidence rate ratios (IRRs) were derived – the admission rate for the hospital relative to the state average rate, taking into account the factors in the model, as well as the size of the hospital's population.[27] The variation between hospital IRRs was measured using the random intercept variance ($\sigma^2$) from the multilevel model, as well as the average relative deviation (ARD) which quantifies, on average, how much these adjusted hospitalisation rates differ from the statewide adjusted admission rate.[28]

Overall IRRs for hospital types were derived by including the hospital category in the model, as a 10% increase in provision of hospital services to the patient's postal area, centred on the mean group value. All analyses were performed in SAS9.4 and MLwiN v2.35.

## Results

Of 267,014 participants in the linked dataset, n=119 were excluded because they had unknown area of residence or incompatible dates in the linked data. Participants in 16 postal areas did not have any hospitalizations during follow-up; the 69 participants residing in these areas were excluded, leaving 266,826 for analysis, over an average follow-up of 3.7 years. Mean age, self-reported health and multi-morbidity of study participants were broadly consistent across remoteness categories (Table 1), although participants in remote areas were slightly younger, with poorer health and a higher number of comorbidities.

The majority of the 30,264 preventable hospitalisations during follow-up were to principal hospitals (31%) with only a small proportion to community (9.1%) and multipurpose (2.6%) facilities (Table 1). However, this pattern was inverted for participants in remote and outer regional areas, with the majority of admissions to community (24.6%) and district hospitals (37.4%). A similar pattern was observed in the 3,167 emergency AMI and 1,550 emergency hip fracture admissions, although with a

For peer review only

smaller proportion of admissions overall to district, community and multipurpose hospitals (data not shown).

There was significant between-hospital variation in preventable hospitalisation, such that each hospital deviated on average 26% from the mean adjusted rate of admission ($\sigma^2$=0.312; standard error [SE]=0.059; ARD=25.6). This variation was much less pronounced for emergency admissions for AMI ($\sigma^2$=0.047; SE=0.026; ARD=9.6) and was not significant for hip fracture ($\sigma^2$=0.015; SE=0.017; ARD=2.9)

Figure 1 shows hospital-level IRRs from the multilevel model, which indicate how each hospital differs from the state average, after adjusting for patient and geographic factors. There was considerable variation in preventable hospitalisation, with 7% of hospitals having significantly higher or lower than average adjusted rates of admission. When stratified by category of hospital, the greatest variation was seen in community, district and multipurpose hospitals, with community hospitals in particular having the highest rates of preventable hospitalisation – up to 4 times the average rate of admission. There were no hospitals with significant deviations from the mean for emergency AMI or hip fracture admissions.

ARDs stratified by hospital category (Figure 1) corroborated these results, with community hospitals having the highest levels of variation in preventable hospitalisation (average 36% difference from the mean), and principal hospitals varying the least (average 21% difference from the mean). There was less variation between all hospital types for emergency AMI or hip fracture admissions than preventable hospitalisations,.

The inclusion of hospital category in the regression models (Table 2) showed significantly higher rates of preventable hospitalisations among people serviced by community (IRR:1.06; 95% CIs:1.02-1.10) and multipurpose (IRR:1.05; 95% CIs:1.01-1.09) than principal hospitals. For emergency AMI admissions, there were significantly higher rates in people serviced by major non-metropolitan (IRR:1.04; 95% CIs:1.02-1.07), and lower rates among people serviced by multipurpose facilities (IRR:0.93; 95% CIs:0.88-0.99). IRRs for all variables in the model are provided in Appendix 3.

A sensitivity analysis categorising preventable hospitalisations as short or long stay admissions (Table 3) found differing patterns of variation by length of stay, with the significantly higher rates of admission for community and multipurpose hospitals restricted to short-stay preventable hospitalisations only.

## Discussion

We found significant variation in rates of preventable hospitalisation between public hospitals, even after adjustment for patient and geographic factors. Our finding was most marked for community and multipurpose hospitals – smaller facilities which provide the majority of services to patients living in regional and remote communities. Given similar variation was not observed for other less-discretionary conditions, major hospitals servicing regional areas, or for admissions with a longer length of stay, our findings indicate a varying propensity to admit patients for preventable hospitalisation among and between categories of hospital facilities.

Our findings do not suggest that preventable hospitalisations should be used as indicator of discretionary admission practice – the effect size was modest and, consistent with prior research,



For peer review only - http://bmjopen.bmj.com/site/about/guidelines.xhtml

the strongest predictors of admission were patient sociodemographic and health characteristics.[9] But while admissions to community and multipurpose hospitals represented only a small proportion (12%) of all preventable hospitalisations, they made up 55% of admissions in remote areas of Australia, where there is both high variability - with over a five-fold variation in rates of preventable hospitalisations[2] - and also the highest rates of admission.[1,2] Accordingly, these differences in admission practices are likely to play an important role in driving geographic variation in the preventable hospitalisations performance indicator. The implications for performance measurement are clear: interpretation of the indicator is complex and factors along the care continuum, including hospitals' propensity to admit, influence variation in admission rates.

There is very little existing evidence about how admissions for preventable hospitalisations vary between hospitals in Australia. One study of major hospitals in NSW reported up to 11-fold and 7-fold variation between hospitals in the proportion of admissions that were for congestive heart failure and chronic obstructive pulmonary disease respectively,[29] and earlier work from the current team found no association between preventable hospitalisations and hospital bed occupancy rates.[26] Importantly, these previous analyses (as with most hospital reporting) excluded community and multipurpose hospitals - the facilities in this study with the strongest patterns of variation. It is difficult to assess causes of between-hospital variation in the context of this analysis. Both differences in hospital roles (e.g. provision of both acute and sub-acute services) and differences in discretionary admission thresholds (e.g. admitting patients for observation to avoid long travel times[15]) could contribute, as well as the provision of community-based services such as hospital in the home[30].

Our study is among a few internationally to provide evidence of a hospital-level difference in propensity to admit patients for preventable hospitalisations,[17,18] and is the first to quantify the extent of this variation. The findings, while not directly applicable to different health care settings, highlight the contextual differences between health systems which should be considered when adopting international performance indicators, as well as the need for localised policy responses tailored to models of care. Use of the preventable hospitalisations indicator beyond its original intent—as a yardstick measure of health system performance[7]—needs to be approached with caution.

The key strength of this study is the use of a large cohort with detailed survey and linked health data. Much inference on preventable hospitalisation is limited either by unmeasured confounders or the use of ecological measures of patient demographics, and estimation of hospital effects can be difficult given the lack of a discrete population denominator. The use of cross-classified multiple membership multilevel models makes this the only study to perform appropriate modelling for each of patient-, area- and hospital-level effects. A limitation is that unexplained hospital variation remained, and we had only limited data on hospital characteristics, so the impact of more complex models of care, such as integrated care programs, has yet to be explored. Generalizability of our findings may also be limited given the older age (45 years and over) and low response rate (18%) of the study cohort, although the considerable size and heterogeneity of the study mean inferences from within-cohort comparisons remain valid.[31]


For peer review only - http://bmjopen.bmj.com/site/about/guidelines.xhtml

## Conclusion

Geographic variation in rates of preventable hospitalisation is determined in part by the hospitals themselves, reflecting different roles of smaller and rural hospitals compared with major and principal referral hospitals. International adoption of the preventable hospitalisations health performance indicator should consider the contextual barriers and facilitators to accessing care in the relevant health system. In Australia, preventable hospitalisations cannot be interpreted simply as a measure of accessibility and quality of primary care.

For peer review only


For peer review only - http://bmjopen.bmj.com/site/about/guidelines.xhtml

## Funding

This study was funded by a National Health and Medical Research Council Partnership Project Grant (#1036858) and by partner agencies the Australian Commission on Safety and Quality in Health Care, the Agency for Clinical Innovation and the NSW Bureau of Health Information. MF is supported by a National Health and Medical Research Council Early Career Fellowship (#1139133). AL receives funding from the Medical Research Council (MC_UU_12017/13) and the Scottish Government Chief Scientist Office (SPHSU13).

## Acknowledgements

The authors would like to thank Federico Girosi and the Australian Primary Health Care Research Institute at the Australian National University for assistance with the data. This research was completed using data collected through the 45 and Up Study (www.saxinstitute.org.au). The 45 and Up Study is managed by the Sax Institute in collaboration with major partner Cancer Council NSW; and partners: National Heart Foundation of Australia (NSW Division); NSW Ministry of Health; NSW Government Family & Community Services – Ageing, Carers and the Disability Council NSW; and the Australian Red Cross Blood Service. We thank the many thousands of people participating in the 45 and Up Study. We thank the Sax Institute, the NSW Ministry of Health and the NSW Register of Births, Deaths and Marriages for allowing access to the data, and the Centre for Health Record Linkage for conducting the probabilistic linkage of records.

## Data availability

The data set used for this study was constructed from pre-existing source data sets (routinely collected data and the 45 and Up Study) with the permission from the custodians of each of these data sets and with specific ethical approval. The data set could potentially be made available to other researchers if they obtain the necessary approvals. Further information on this process can be obtained from the 45 and Up Study (45andUp.research@saxinstitute.org.au) and the NSW Centre for Health Record Linkage (cherel.mail@moh.health.nsw.gov.au).

## Author contributions

MOF conceived the project, undertook the literature review, performed data analysis and drafted the manuscript. LRJ and AHL provided guidance and interpretation. All three authors edited, reviewed and approved the final manuscript. LRJ conceived the APHID study

## Competing interests

None declared



For peer review only - http://bmjopen.bmj.com/site/about/guidelines.xhtml

## References


1 Australian Commission on Safety and Quality in Health Care and Australian Institute of Health and Welfare.. The Second Australian Atlas of Healthcare Variation. (ACSQHC, Sydney, 2017).

2 National Health Performance Authority. Healthy Communities: Potentially preventable hospitalisations in 2013–14. (NHPA, Sydney, 2015).

3 Torio, C. M., Elixhauser, A. & Andrews, R. M. Trends in potentially preventable hospital admissions among adults and children, 2005–2010. (Agency for Healthcare Research and Quality, Rockville, MD, 2013).

4 Australian Institute of Health and Welfare. Admitted patient care 2014–15: Australian hospital statistics. (AIHW, Canberra, 2016).

5 Baker, D. W. & Chassin, M. R. Holding Providers Accountable for Health Care Outcomes. *Annals of Internal Medicine* **167**, 418-423, doi:10.7326/M17-0691 (2017).

6 Giuffrida, A., Gravelle, H. & Roland, M. Measuring quality of care with routine data: avoiding confusion between performance indicators and health outcomes. *Brit Med J* **319**, 94-98, doi:10.1136/bmj.319.7202.94 (1999).

7 Billings, J. *et al.* Impact of socioeconomic status on hospital use in New York City. *Health Affairs (Project Hope)* **12**, 162-173 (1993).

8 Weissman, J. S., Gatsonis, C. & Epstein, A. M. Rates of avoidable hospitalization by insurance status in Massachusetts and Maryland. *JAMA* **268**, 2388-2394 (1992).

9 Falster, M. O. *et al.* Sociodemographic and health characteristics, rather than primary care supply, are major drivers of geographic variation in preventable hospitalizations in Australia. *Med Care* **53**, 436-445, doi:10.1097/MLR.0000000000000342 (2015).

10 Roos, L. L., Walld, R., Uhanova, J. & Bond, R. Physician visits, hospitalizations, and socioeconomic status: ambulatory care sensitive conditions in a canadian setting. *Health Serv Res* **40**, 1167-1185, doi:10.1111/j.1475-6773.2005.00407.x (2005).

11 Trachtenberg, A. J., Dik, N., Chateau, D. & Katz, A. Inequities in ambulatory care and the relationship between socioeconomic status and respiratory hospitalizations: a population-based study of a Canadian city. *Annals of Family Medicine* **12**, 402-407, doi:10.1370/afm.1683 (2014).

12 Dean, N. C. *et al.* Hospital admission decision for patients with community-acquired pneumonia: variability among physicians in an emergency department. *Ann Emerg Med* **59**, 35-41, doi:10.1016/j.annemergmed.2011.07.032 (2012).

13 Fisher, E. S. *et al.* Associations among hospital capacity, utilization, and mortality of US Medicare beneficiaries, controlling for sociodemographic factors. *Health Serv Res* **34**, 1351-1362 (2000).

14 Wennberg, J., McPherson, K. & Goodman, D. in *Medical Practice Variations* (eds Ana Johnson & Thérèse Stukel) 1-25 (Springer US, 2016).

15 Culliton, G. Large variance in hospitals' 'propensity to admit', in *Irish Medical Times* (2016).

16 National Health Performance Authority. Healthy Communities: Use of emergency department and GP services in 2013-14. (NHPA, Sydney, 2016).

17 O'Cathain, A. *et al.* Hospital characteristics affecting potentially avoidable emergency admissions: national ecological study. *Health Services Management Research* **26**, 110-118, doi:10.1177/0951484814525357 (2013).

18 Zhan, C., Miller, M. R., Wong, H. & Meyer, G. S. The effects of HMO penetration on preventable hospitalizations. *Health Serv Res* **39**, 345-361, doi:10.1111/j.1475-6773.2004.00231.x (2004).

19 Basu, J., Friedman, B. & Burstin, H. Primary care, HMO enrollment, and hospitalization for ambulatory care sensitive conditions: a new approach. *Med Care* **40**, 1260-1269, doi:10.1097/01.MLR.0000036409.56436.ED (2002).




For peer review only - http://bmjopen.bmj.com/site/about/guidelines.xhtml

20 Berlin, C., Busato, A., Rosemann, T., Djalali, S. & Maessen, M. Avoidable hospitalizations in Switzerland: a small area analysis on regional variation, density of physicians, hospital supply and rurality. *BMC Health Serv Res* **14**, 289, doi:info:pmid/24992827 (2014).

21 Banks, E. *et al.* Cohort profile: the 45 and up study. *International Journal of Epidemiology* **37**, 941-947, doi:10.1093/ije/dym184 (2008).

22 AIHW. *National Healthcare Agreement: PI 22-Selected potentially preventable hospitalisations, 2012*, <http://meteor.aihw.gov.au/content/index.phtml/itemId/443687> (2012).

23 Swerissen, H., Duckett, S. & Wright, J. Chronic failure in primary care. Report No. 2016-2, (Grattan Institute, 2016).

24 Mazumdar, S., Konings, P., Butler, D. & McRae, I. S. General practitioner (family physician) workforce in Australia: comparing geographic data from surveys, a mailing list and medicare. *BMC Health Serv Res* **13**, 343, doi:10.1186/1472-6963-13-343 (2013).

25 NSW Health. NSW Health Services Comparison Data Book 2008/2009. (Demand and Performance Evaluation, NSW Health, North Sydney, NSW, 2010).

26 Falster, M. O., Jorm, L. R. & Leyland, A. H. Using Weighted Hospital Service Area Networks to Explore Variation in Preventable Hospitalization. *Health Serv Res* **53**, 3148-3169, doi:10.1111/1475-6773.12777 (2018).

27 Merlo, J., Chaix, B., Yang, M., Lynch, J. & Rastam, L. A brief conceptual tutorial of multilevel analysis in social epidemiology: linking the statistical concept of clustering to the idea of contextual phenomenon. *J Epidemiol Community Health* **59**, 443-449, doi:10.1136/jech.2004.023473 (2005).

28 Tarkiainen, L., Martikainen, P., Laaksonen, M. & Leyland, A. H. Comparing the effects of neighbourhood characteristics on all-cause mortality using two hierarchical areal units in the capital region of Helsinki. *Health Place* **16**, 409-412, doi:10.1016/j.healthplace.2009.10.008 (2010).

29 Bureau of Health Information. Chronic disease care: A piece of the picture. (BHI, Sydney, Australia, 2011).

30 NSW Ministry of Health. Hospital in the Home Guideline. (NSW Ministry of Health, North Sydney, 2013).

31 Mealing, N. M. *et al.* Investigation of relative risk estimates from studies of the same population with contrasting response rates and designs. *BMC Medical Research Methodology* **10**, 26, doi:10.1186/1471-2288-10-26 (2010).


For peer review only



For peer review only - http://bmjopen.bmj.com/site/about/guidelines.xhtml

# Tables and Figures

***Table 1: Cohort characteristics at baseline, and number of preventable hospitalisations during follow-up, by remoteness of area of residence***

| | Total | By remoteness category of residence | | | |
|---|---|---|---|---|---|
| | | Major cities | Inner regional | Outer regional | Remote |
| **Cohort characteristics** | | | | | |
| N | 266,826 | 119,496 | 94,568 | 47,438 | 5,324 |
| Age (mean) | 62.7 | 63.4 | 62.4 | 62.2 | 60.7 |
| Age (IQR) | 53.6-70.4 | 53.6-71.9 | 53.8-69.7 | 53.7-69.4 | 52.0-67.8 |
| % Female | 53.6 | 52.4 | 54.7 | 54.3 | 55.5 |
| % fair/poor self-rated health | 13.7 | 13.9 | 13.4 | 13.7 | 16.1 |
| % with >3 comorbidities | 7.4 | 7.3 | 7.5 | 7.2 | 8.0 |
| **Preventable hospitalisations** | | | | | |
| Number of admissions | 30,264 | 12,512 | 10,161 | 6512 | 1079 |
| Admissions to hospital type (%) | | | | | |
| - Principal | 9398 (31.0) | 7506 (60.0) | 1600 (15.7) | 255 (3.9) | 37 (3.4) |
| - Major metropolitan | 4172 (13.8) | 3321 (26.5) | 787 (7.7) | 61 (0.9) | 3 (0.3) |
| - Major non-metropolitan | 6443 (21.3) | 560 (4.5) | 3933 (38.7) | 1872 (28.7) | 78 (7.2) |
| - District | 6715 (22.2) | 804 (6.4) | 3070 (30.2) | 2468 (37.9) | 373 (34.6) |
| - Community | 2760 (9.1) | 278 (2.2) | 611 (6.1) | 1491 (22.9) | 380 (35.2) |
| - Multipurpose | 776 (2.6) | 43 (0.3) | 160 (1.6) | 365 (5.6) | 208 (19.3) |

For peer review only

*Table 2: Incidence rate ratio (IRR) of hospital category for preventable hospitalisation and emergency admissions for acute myocardial infarction (AMI) and hip fracture*

| | Preventable hospitalisations | | AMI (emergency) | | Hip fracture (emergency) | |
|---|---|---|---|---|---|---|
| | IRR | (95% CIs) | IRR | (95% CIs) | IRR | (95% CIs) |
| Hospital category | | | | | | |
| Principal | 1.00 | (ref) | 1.00 | (ref) | 1.00 | (ref) |
| Major metropolitan | 0.99 | (0.95 – 1.03) | 1.02 | (0.99 – 1.05) | 1.02 | (0.99 – 1.05) |
| Major non-metropolitan | 1.01 | (0.97 – 1.04) | 1.04 | (1.02 – 1.07) | 0.99 | (0.96 – 1.02) |
| District | 1.02 | (0.99 – 1.06) | 1.00 | (0.97 – 1.03) | 0.99 | (0.96 – 1.02) |
| Community | 1.06 | (1.02 – 1.10) | 0.97 | (0.93 – 1.01) | 0.96 | (0.91 – 1.01) |
| Multipurpose | 1.05 | (1.01 – 1.09) | 0.93 | (0.88 – 0.99) | 1.02 | (0.94 – 1.09) |

For peer review only


For peer review only - http://bmjopen.bmj.com/site/about/guidelines.xhtml

***Table 3: Average relative deviation (ARD) and Incidence rate ratio (IRR) by hospital category for rates of preventable hospitalisation, separated as short-stay (0-2 days length of stay) and long-stay (>2 days length of stay) admissions***

| | Short stay (0-2 days length of stay) | | | Long stay (>2 days length of stay) | | |
|---|---|---|---|---|---|---|
| | ARD | IRR | (95% CIs) | ARD | IRR | (95% CIs) |
| Hospital category | | | | | | |
| Principal | 17.9 | 1.00 | (ref) | 14.6 | 1.00 | (ref) |
| Major metropolitan | 25.5 | 0.99 | (0.95 – 1.02) | 25.9 | 1.00 | (0.97 – 1.03) |
| Major non-metropolitan | 22.7 | 1.02 | (0.98 – 1.05) | 11.3 | 0.99 | (0.96 – 1.02) |
| District | 30.4 | 1.02 | (0.99 – 1.05) | 24.3 | 0.98 | (0.95 – 1.00) |
| Community | 17.5 | 1.04 | (1.01 – 1.07) | 25.7 | 1.02 | (0.99 – 1.05) |
| Multipurpose | 24.3 | 1.04 | (1.00 – 1.08) | 11.6 | 0.99 | (0.95– 1.03) |

For peer review only

***Figure 1: Hospital-specific incidence rate ratios from the mean adjusted rate of admission, for preventable hospitalisation and emergency admissions for acute myocardial infarction (AMI) and hip fracture, overall and stratified by hospital category***

ARD = average relative deviation. Red and blue markers indicate hospitals with significantly higher and lower rates of admission respectively. Adjusted for patient sociodemographic and health factors, remoteness and supply of GP services in area of residence

For peer review only


For peer review only - http://bmjopen.bmj.com/site/about/guidelines.xhtml

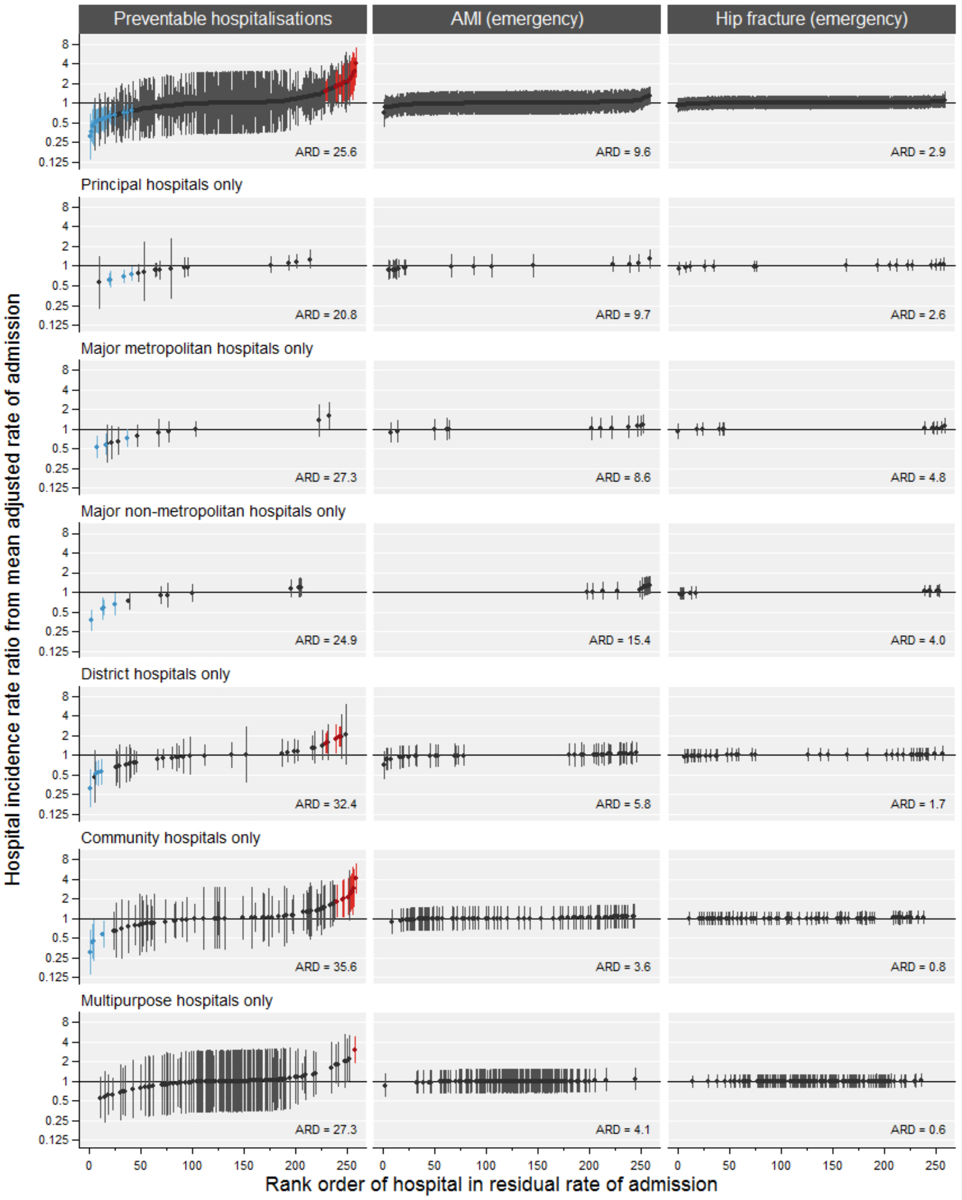


Hospital-specific incidence rate ratios from the mean adjusted rate of admission, for preventable hospitalisation and emergency admissions for acute myocardial infarction (AMI) and hip fracture, overall and stratified by hospital category

301x377mm (72 x 72 DPI)

# Online appendices

## Appendix 1:

ICD-10-AM codes for identifying hospital outcomes.

| Category | ICD-10-AM diagnosis and procedure codes |
|---|---|
| **Preventable hospitalisations** | |
| Angina | I20, I24.0, I24.8, I24.9 as principal diagnosis only, exclude cases with procedure codes not in blocks [1820] to [2016] |
| Asthma | J45, J46 as principal diagnosis only |
| Chronic obstructive pulmonary disease (COPD) | J20, J41, J42, J43, J44, J47 as principal diagnosis only, J20 only with additional diagnoses of J41, J42, J43,J44, J47 |
| Congestive cardiac failure | I50, I11.0, J81 as principal diagnosis only, exclude cases with the following procedure codes: 33172-00, 35304-00, 35305-00, 35310-02, 35310-00, 38281-11, 38281-07, 38278-01, 38278-00, 38281-02, 38281-01, 38281-00, 38256-00, 38278-03, 38284-00, 38284-02, 38521-09, 38270-01, 38456-19, 38456-15, 38456-12, 38456-11, 38456-10, 38456-07, 38456-01, 38470-00, 38475-00, 38480-02, 38480-01, 38480-00, 38488-06, 38488-04, 38489-04, 38488-02, 38489-03, 38487-00, 38489-02, 38488-00, 38489-00, 38490-00, 38493-00, 38497-04, 38497-03, 38497-02, 38497-01, 38497-00, 38500-00, 38503-00, 38505-00, 38521-04, 38606-00, 38612-00, 38615-00, 38653-00, 38700-02, 38700-00, 38739-00, 38742-02, 38742-00, 38745-00, 38751-02, 38751-00, 38757-02, 38757-01, 38757-00, 90204-00, 90205-00, 90219-00, 90224-00, 90214-00, 90214-02. |
| Diabetes complications | E10–E14.9 as principal diagnoses, and E10–E14.9 as additional diagnoses where the principal diagnosis was: hypersmolarity (E87.0), acidosis (E87.2), transient ischaemic attack (G45), nerve disorders and neuropathies (G50–G64), cataracts and lens disorders (H25–H28), retinal disorders (H30–H36), glaucoma (H40–H42), myocardial infarction (I21–I22), other coronary heart diseases (I20, I23–I25), heart failure (I50), stroke and sequelae (I60–I64, I69.0–I69.4), peripheral vascular disease (I70–I74), gingivitis and periodontal disease (K05), kidney diseases including end-stage renal disease (N00–N29), and renal dialysis (Z49) |
| Hypertension | I10, I11.9 as principal diagnosis only, exclude cases with procedure codes according to the list of procedures excluded from the Congestive cardiac failure category above. |
| Iron deficiency anaemia | D50.1, D50.8, D50.9 as principal diagnosis only. |
| Nutritional deficiencies | E40, E41, E42, E43, E55.0, E64.3 as principal diagnosis only. |
| Rheumatic heart disease | I00 to I09 as principal diagnosis only. (Note: includes acute rheumatic fever) |
| Appendicitis with generalised peritonitis | K35.0 in any diagnosis field |
| Cellulitis | L03, L04, L08, L88, L98.0, L98.3 as principal diagnosis only, exclude cases with any procedure except those in blocks 1820 to 2016 or if procedure is 30216-02, 30676-00, 30223-02, 30064-00, 34527-01, 34527-00, 90661-00 and this is the only listed procedure |
| Convulsions and epilepsy | G40, G41, O15, R56 as principal diagnosis only |
| Dehydration and gastroenteritis | A09.9, E86, K52.2, K52.8, K52.9 as principal diagnosis only. |
| Dental conditions | K02, K03, K04, K05, K06, K08, K09.8, K09.9, K12, K13 as principal diagnosis only. |
| Ear, nose and throat infections | H66, H67, J02, J03, J06, J31.2 as principal diagnosis only. |
| Gangrene | R02 in any diagnosis field |
| Pelvic inflammatory disease | N70, N73, N74 as principal diagnosis only. |
| Perforated/bleeding ulcer | K25.0, K25.1, K25.2, K25.4, K25.5, K25.6, K26.0, K26.1, K26.2, K26.4, K26.5, K26.6, K27.0, K27.1, K27.2, K27.4, K27.5, K27.6, K28.0, K28.1, K28.2, K28.4, K28.5, K28.6 as principal diagnosis only. |
| Pyelonephritis | N10, N11, N12, N13.6, N39.0 as principal diagnosis only. |
| Influenza and pneumonia | J10, J11, J13, J14, J15.3, J15.4, J15.7, J15.9, J16.8, J18.1, J18.8 in any diagnosis field, excludes cases with additional diagnosis of D57 (sickle-cell disorders) and people under 2 months |
| Other vaccine-preventable conditions | A35, A36, A37, A80, B05, B06, B16.1, B16.9, B18.0, B18.1, B26, G00.0, M01.4 in any diagnosis field |
| Acute myocardial infarction (AMI) | I21 |
| Hip fracture | S72.0, S72.1, S72.2 |

For peer review only

# Appendix 2:

Hospital categories, corresponding peer groups from the *NSW Health Services Comparison Data Book 2008/2009*, and number all cause admissions during follow-up.

| Hospital Peer Group | Description | Admissions |
|---|---|---|
| **Principal** | | |
| A1a Principal Referral Group A | Acute hospitals, treating 25,000 or more acute casemix weighted separations per annum, with an average cost weight greater than 1 and having more than 1 specialty service. | 76,193 |
| A1b Principal Referral Group B | Acute hospitals, treating 25,000 or more acute casemix weighted separations per annum, with an average cost weight greater than 1 and 1 or fewer specialty services. | 28,424 |
| A2 Paediatric Specialist | Establishments where the primary role is to provide specialist acute care services for children. | - |
| A3 Ungrouped Acute | Establishments whose primary role is the provision of acute services of a specialised nature for which there is insufficient peers to form additional peer groups. | 6,284 |
| **Major metropolitan** | | |
| B1 Major Metropolitan: | Acute hospitals, treating 10,000 or more acute casemix weighted separations per annum, but having less than 25,000 acute casemix weighted separations or an average casemix weight of less than 1. | 30,512 |
| **Major non-metropolitan** | | |
| B2 Major Non-Metropolitan: | Acute hospitals treating 10,000 or more acute casemix weighted separations per annum that are located in rural areas providing acute specialist and referral services for a catchment population from a large geographical area. | 68,460 |
| **District** | | |
| C1 District Group 1 | Acute hospitals, treating 5,000 or more, but less than 10,000 acute casemix weighted separations per annum. | 27,671 |
| C2 District Group 2 | Acute hospitals, treating 2,000 or more, but less than 5,000 acute casemix weighted separations per annum, plus acute hospitals treating less than 2,000 acute casemix weighted separations per annum but with more than 2,000 separations per annum. | 29,470 |
| **Community** | | |
| D1a Community Acute with Surgery | Acute hospitals, treating less than 2,000 acute casemix weighted separations per annum, and less than 2,000 acute separations per annum, with less than 40% nonacute and outlier bed days of total bed days and greater than 2% of their acute weighted separations being surgical. | 6,352 |
| D1b Community Acute without Surgery | Acute hospitals, treating less than 2,000 acute casemix weighted separations per annum, and less than 2,000 acute separations per annum, with less than 40% nonacute and outlier bed days of total bed days, and less than 2% of their acute weighted separations being surgical. | 3,620 |
| D2 Community Non-Acute | Non-acute hospitals, treating less than 2,000 acute casemix weighted separations per annum, and less than 2,000 acute separations per annum, with more than 40% nonacute and outlier bed days of total bed days. | 3,061 |
| **Multi-purpose** | | |
| F1 Psychiatric | Establishments devoted primarily to the treatment and care of inpatients with psychiatric, mental or behavioural disorders. Centres of non-acute treatment of drug dependence, developmental and intellectual disability are not included here. This group also excludes institutions mainly providing living quarters or day care. | 195 |
| F2 Nursing Homes | Establishments which provide long-term care involving regular base nursing care to chronically ill, frail, disabled or convalescent persons or senile inpatients. They must be approved by the Commonwealth Department of Health and Family Services and /or licensed by the State, or controlled by government departments. | 184 |
| F3 Multi-Purpose Services | Multi-Purpose Services (MPSs) which provide integrated acute health, nursing home, hostel, community health and aged care services under one organisational structure, as agreed between the Commonwealth and State Governments. MPSs provide a range of services which are negotiated with the community, the service providers and the relevant Departments. | 4,493 |
| F4 Sub Acute | Establishments that primarily provide sub-acute services, but are not specialist palliative care or specialist rehabilitation establishments. | 2,588 |
| F5 Palliative Care | Establishments with a specific function of providing palliative care to terminally ill patients. | 86 |
| F6 Rehabilitation | Establishments with a primary role in providing services to persons with an impairment, disability or handicap where the primary goal is improvement in functional status. | 34 |
| F7 Mothercraft | Establishments where the primary role is to help mothers acquire mothercraft skills in an inpatient setting. | 5 |
| F8 Ungrouped Non-Acute | Establishments whose primary role is the provision of non-acute services, but for which there are insufficient peers to form an addition peer group. Limited comparisons can be made within this peer group and with other non-acute facilities. | 328 |

For peer review only

# Appendix 3:

Incidence rate ratio (IRR) of patient-, area- and hospital-level factors from cross-classified multiple membership multilevel Poisson models on preventable hospitalisations and emergency admissions for acute myocardial infarction (AMI) and hip fracture

| | Preventable hospitalisation | | AMI (emergency) | | Hip fracture (emergency) | |
|---|---|---|---|---|---|---|
| | IRR | (95% CIs) | IRR | (95% CIs) | IRR | (95% CIs) |
| **Patient-level factors** | | | | | | |
| Age | | | | | | |
| 45-54 years | 1.00 | (ref) | 1.00 | (ref) | 1.00 | (ref) |
| 55-64 years | 1.23 | (1.18 - 1.29) | 1.71 | (1.49 - 1.97) | 2.25 | (1.52 - 3.33) |
| 65-74 years | 1.72 | (1.64 - 1.81) | 2.39 | (2.05 - 2.80) | 7.12 | (4.89 - 10.4) |
| 75-84 years | 2.57 | (2.44 - 2.70) | 4.23 | (3.61 - 4.96) | 24.7 | (17.1 - 35.7) |
| 85 years and over | 3.40 | (3.20 - 3.61) | 7.64 | (6.35 - 9.18) | 49.2 | (33.7 - 71.6) |
| Sex | | | | | | |
| Males | 1.00 | (ref) | 1.00 | (ref) | 1.00 | (ref) |
| Females | 0.72 | (0.70 - 0.74) | 0.42 | (0.39 - 0.46) | 1.36 | (1.21 - 1.53) |
| Highest level of education | | | | | | |
| Did not complete high school | 1.00 | (ref) | 1.00 | (ref) | 1.00 | (ref) |
| High school or equivalent | 0.93 | (0.91 - 0.96) | 0.92 | (0.85 - 0.99) | 1.02 | (0.91 - 1.15) |
| University or higher | 0.86 | (0.83 - 0.90) | 0.78 | (0.69 - 0.88) | 1.16 | (0.98 - 1.38) |
| Unknown / missing | 1.13 | (1.07 - 1.20) | 0.95 | (0.77 - 1.16) | 0.90 | (0.68 - 1.18) |
| Marital status | | | | | | |
| Single | 1.00 | (ref) | 1.00 | (ref) | 1.00 | (ref) |
| Married / partnered | 1.19 | (1.13 - 1.25) | 1.09 | (0.93 - 1.28) | 1.31 | (1.05 - 1.63) |
| Widowed / divorced / separated | 1.17 | (1.13 - 1.20) | 1.19 | (1.10 - 1.30) | 1.14 | (1.01 - 1.28) |
| Unknown / missing | 1.22 | (1.08 - 1.37) | 0.79 | (0.50 - 1.25) | 0.57 | (0.25 - 1.29) |
| Annual household income | | | | | | |
| <$10,000 | 1.00 | (ref) | 1.00 | (ref) | 1.00 | (ref) |
| $10,000 - $29,999 | 0.89 | (0.86 - 0.92) | 0.87 | (0.76 - 0.99) | 1.04 | (0.87 - 1.25) |
| $30,000 - $49,000 | 0.80 | (0.76 - 0.84) | 0.89 | (0.76 - 1.04) | 0.80 | (0.62 - 1.02) |
| $50,000 - $69,999 | 0.75 | (0.70 - 0.80) | 0.85 | (0.70 - 1.03) | 0.88 | (0.63 - 1.21) |
| $70,000 or more | 0.65 | (0.61 - 0.69) | 0.75 | (0.62 - 0.90) | 0.85 | (0.63 - 1.16) |
| Not specified | 0.94 | (0.90 - 0.98) | 1.04 | (0.90 - 1.20) | 1.05 | (0.86 - 1.28) |
| Unknown / missing | 1.13 | (1.07 - 1.18) | 1.14 | (0.97 - 1.34) | 1.15 | (0.93 - 1.43) |
| Employment status | | | | | | |
| Not working | 1.00 | (ref) | 1.00 | (ref) | 1.00 | (ref) |
| Part time | 0.82 | (0.79 - 0.86) | 0.90 | (0.78 - 1.04) | 0.63 | (0.47 - 0.84) |
| Full time | 0.84 | (0.80 - 0.88) | 1.16 | (1.01 - 1.33) | 0.90 | (0.67 - 1.19) |
| Missing / unknown | 0.93 | (0.85 - 1.01) | 1.03 | (0.78 - 1.38) | 0.91 | (0.64 - 1.28) |
| Language spoken at home | | | | | | |
| English | 1.00 | (ref) | 1.00 | (ref) | 1.00 | (ref) |
| Other | 0.92 | (0.88 - 0.96) | 0.99 | (0.87 - 1.12) | 1.04 | (0.88 - 1.23) |
| Health insurance status | | | | | | |
| Private with extras | 1.00 | (ref) | 1.00 | (ref) | 1.00 | (ref) |
| Private, no extras | 1.03 | (0.99 - 1.08) | 1.05 | (0.93 - 1.18) | 0.93 | (0.79 - 1.09) |
| Department of Veterans Affairs | 1.28 | (1.21 - 1.36) | 1.08 | (0.91 - 1.28) | 1.11 | (0.91 - 1.35) |
| Health Care Card | 1.60 | (1.54 - 1.65) | 1.42 | (1.29 - 1.57) | 0.94 | (0.82 - 1.08) |


For peer review only - http://bmjopen.bmj.com/site/about/guidelines.xhtml

| | Preventable hospitalisation | | AMI (emergency) | | Hip fracture (emergency) | |
|---|---|---|---|---|---|---|
| | IRR | (95% CIs) | IRR | (95% CIs) | IRR | (95% CIs) |
| None | 1.50 | (1.44 - 1.55) | 1.26 | (1.13 - 1.41) | 0.93 | (0.78 - 1.10) |
| Number of people can depend on | | | | | | |
| 0 people | 1.00 | (ref) | 1.00 | (ref) | 1.00 | (ref) |
| 1-4 people | 1.09 | (1.04 - 1.14) | 0.92 | (0.80 - 1.06) | 1.20 | (0.96 - 1.49) |
| 5-10 people | 1.07 | (1.02 - 1.12) | 0.92 | (0.80 - 1.07) | 1.18 | (0.94 - 1.47) |
| 11 or more people | 1.20 | (1.14 - 1.27) | 0.89 | (0.75 - 1.06) | 1.15 | (0.89 - 1.50) |
| Unknown / missing | 1.16 | (1.10 - 1.24) | 0.99 | (0.82 - 1.20) | 1.35 | (1.04 - 1.77) |
| Positive health seeking behaviours [a] | | | | | | |
| 0 health behaviours | 0.96 | (0.86 - 1.07) | 1.02 | (0.72 - 1.43) | 1.31 | (0.77 - 2.23) |
| 1 health behaviour | 1.00 | (ref) | 1.00 | (ref) | 1.00 | (ref) |
| 2 health behaviours | 0.89 | (0.86 - 0.93) | 0.89 | (0.80 - 1.00) | 0.76 | (0.65 - 0.89) |
| 3 health behaviours | 0.78 | (0.75 - 0.81) | 0.76 | (0.68 - 0.85) | 0.65 | (0.55 - 0.77) |
| 4 health behaviours | 0.76 | (0.72 - 0.80) | 0.75 | (0.64 - 0.88) | 0.55 | (0.43 - 0.70) |
| BMI | | | | | | |
| Underweight | 1.13 | (1.08 - 1.17) | 1.01 | (0.88 - 1.15) | 1.10 | (0.96 - 1.27) |
| Healthy weight | 1.00 | (ref) | 1.00 | (ref) | 1.00 | (ref) |
| Overweight | 0.94 | (0.91 - 0.97) | 0.95 | (0.87 - 1.03) | 0.57 | (0.50 - 0.64) |
| Obese | 1.00 | (0.96 - 1.03) | 1.00 | (0.90 - 1.10) | 0.35 | (0.29 - 0.42) |
| Unknown / missing | 1.20 | (1.10 - 1.32) | 1.10 | (0.80 - 1.50) | 0.69 | (0.41 - 1.16) |
| Self-rated health | | | | | | |
| Excellent | 1.00 | (ref) | 1.00 | (ref) | 1.00 | (ref) |
| Very good | 1.22 | (1.13 - 1.30) | 1.21 | (1.03 - 1.43) | 0.86 | (0.67 - 1.10) |
| Good | 1.60 | (1.50 - 1.71) | 1.32 | (1.12 - 1.55) | 1.04 | (0.81 - 1.33) |
| Fair | 2.58 | (2.40 - 2.76) | 1.59 | (1.33 - 1.90) | 1.18 | (0.91 - 1.54) |
| Poor | 4.10 | (3.79 - 4.43) | 1.84 | (1.47 - 2.32) | 1.66 | (1.20 - 2.29) |
| Unknown / missing | 2.27 | (2.09 - 2.46) | 1.37 | (1.09 - 1.72) | 1.04 | (0.77 - 1.42) |
| Multi-morbid conditions [b] | | | | | | |
| No conditions | 1.00 | (ref) | 1.00 | (ref) | 1.00 | (ref) |
| 1 condition | 1.35 | (1.31 - 1.40) | 1.20 | (1.09 - 1.32) | 1.00 | (0.87 - 1.14) |
| 2 conditions | 1.98 | (1.91 - 2.05) | 1.45 | (1.30 - 1.62) | 0.90 | (0.77 - 1.05) |
| 3 or more conditions | 2.71 | (2.60 - 2.82) | 2.26 | (2.01 - 2.55) | 1.06 | (0.90 - 1.26) |
| Functional limitations [c] | | | | | | |
| No limitation | 1.00 | (ref) | 1.00 | (ref) | 1.00 | (ref) |
| Minor limitations | 1.04 | (0.97 - 1.10) | 0.97 | (0.84 - 1.14) | 0.83 | (0.60 - 1.16) |
| Mild limitation | 1.25 | (1.18 - 1.32) | 1.04 | (0.91 - 1.19) | 1.13 | (0.86 - 1.48) |
| Moderate limitation | 1.56 | (1.48 - 1.64) | 1.17 | (1.03 - 1.34) | 1.86 | (1.45 - 2.38) |
| Severe limitation | 2.36 | (2.24 - 2.48) | 1.49 | (1.30 - 1.71) | 2.83 | (2.21 - 3.63) |
| Unknown / missing | 1.71 | (1.61 - 1.80) | 1.24 | (1.07 - 1.44) | 1.75 | (1.36 - 2.26) |
| Psychological distress [d] | | | | | | |
| Low distress | 1.00 | (ref) | 1.00 | (ref) | 1.00 | (ref) |
| Moderate distress | 1.03 | (0.99 - 1.06) | 1.01 | (0.91 - 1.12) | 1.03 | (0.88 - 1.20) |
| High distress | 0.99 | (0.95 - 1.03) | 1.08 | (0.92 - 1.26) | 1.10 | (0.87 - 1.39) |
| Very high distress | 0.99 | (0.94 - 1.06) | 0.88 | (0.69 - 1.12) | 1.18 | (0.83 - 1.66) |
| Unknown / missing | 1.09 | (1.03 - 1.15) | 1.31 | (1.12 - 1.54) | 1.16 | (0.95 - 1.42) |
| **Area-level factors** | | | | | | |
| Remoteness of residence | | | | | | |

For peer review only

| | Preventable hospitalisation | | AMI (emergency) | | Hip fracture (emergency) | |
|---|---|---|---|---|---|---|
| | IRR | (95% CIs) | IRR | (95% CIs) | IRR | (95% CIs) |
| Major city | 1.00 | (ref) | 1.00 | (ref) | 1.00 | (ref) |
| Inner regional | 0.95 | (0.88 - 1.02) | 1.05 | (0.91 - 1.22) | 1.13 | (0.95 - 1.33) |
| Outer regional | 1.01 | (0.88 - 1.15) | 1.08 | (0.87 - 1.34) | 1.06 | (0.81 - 1.38) |
| Remote / very remote | 1.24 | (0.97 - 1.58) | 0.95 | (0.61 - 1.48) | 0.84 | (0.44 - 1.62) |
| Full-time workload equivalent GPs | | | | | | |
| Quintile 1 (2.64-6.90 GPs) | 1.00 | (ref) | 1.00 | (ref) | 1.00 | (ref) |
| Quintile 2 (6.91-7.60 GPs) | 0.89 | (0.78 - 1.02) | 0.88 | (0.73 - 1.07) | 1.01 | (0.80 - 1.29) |
| Quintile 3 (7.63-8.64 GPs) | 0.95 | (0.84 - 1.08) | 0.89 | (0.74 - 1.08) | 1.10 | (0.87 - 1.39) |
| Quintile 4 (8.65-9.94 GPs) | 0.92 | (0.79 - 1.06) | 0.88 | (0.72 - 1.07) | 1.04 | (0.82 - 1.32) |
| Quintile 5 (9.95-13.3 GPs) | 1.02 | (0.87 - 1.19) | 0.91 | (0.74 - 1.12) | 0.96 | (0.74 - 1.23) |
| **Hospital-level factors** | | | | | | |
| Hospital category | | | | | | |
| Principal | 1.00 | (ref) | 1.00 | (ref) | 1.00 | (ref) |
| Major metropolitan | 0.99 | (0.95 - 1.03) | 1.02 | (0.99 - 1.05) | 1.02 | (0.99 - 1.05) |
| Major non-metropolitan | 1.01 | (0.97 - 1.04) | 1.04 | (1.02 - 1.07) | 0.99 | (0.96 - 1.02) |
| District | 1.02 | (0.99 - 1.06) | 1.00 | (0.97 - 1.03) | 0.99 | (0.96 - 1.02) |
| Community | 1.06 | (1.02 - 1.10) | 0.97 | (0.93 - 1.01) | 0.96 | (0.91 - 1.01) |
| Multipurpose | 1.05 | (1.01 - 1.09) | 0.93 | (0.88 - 0.99) | 1.02 | (0.94 - 1.09) |
| **Random effects** | | | | | | |
| Residual random effect (SE) | | | | | | |
| Hospital-level | 0.276 | (0.056) | 0.010 | (0.013) | 0.024 | (0.022) |
| Area-level | 0.061 | (0.011) | 0.050 | (0.015) | 0.013 | (0.011) |

[a] Healthy behaviours, of non-smoking status, safe level of alcohol consumption (<14 drinks per week), at least 2.5 hours of intensity-weighted physical activity per week, and meeting dietary guidelines for daily fruit (2 serves) and vegetable (5 serves) consumption

[b] Of self-reported heart disease, high blood pressure, stroke, diabetes, blood clot, asthma, Parkinson's disease, and any cancer except skin cancer.

[c] Measured using the Medical Outcome Study physical functioning scale.

[d] Measured using the K10 scale.



For peer review only - http://bmjopen.bmj.com/site/about/guidelines.xhtml

BMJ Open

# Do hospitals influence geographic variation in admission for preventable hospitalisation? A data linkage study in New South Wales, Australia

| | |
|---|---|
| Journal: | *BMJ Open* |
| Manuscript ID | bmjopen-2018-027639.R1 |
| Article Type: | Research |
| Date Submitted by the Author: | 17-Dec-2018 |
| Complete List of Authors: | Falster, Michael; University of New South Wales, Centre for Big Data Research in Health<br>Leyland, Alastair; MRC/CSO Social and Public Health Sciences Unit, University of Glasgow,<br>Jorm, Louisa; University of New South Wales, Centre for Big Data Research in Health |
| <b>Primary Subject Heading</b>: | Health services research |
| Secondary Subject Heading: | Epidemiology |
| Keywords: | PRIMARY CARE, Preventable hospitalisations, Ambulatory care sensitive conditions, Multilevel modelling |
| | |

SCHOLARONE™
Manuscripts

# Title

Do hospitals influence geographic variation in admission for preventable hospitalisation? A data linkage study in New South Wales, Australia

# Authors

Michael O Falster, PhD[1]; Alastair H Leyland, PhD[2]; Louisa R Jorm, PhD[1];

[1] Centre for Big Data Research in Health, UNSW Sydney, Australia

[2] MRC/CSO Social and Public Health Sciences Unit, University of Glasgow, Glasgow UK

# Corresponding author

Dr Michael O. Falster

Centre for Big Data Research in Health, UNSW Sydney

Level 1, AGSM Building (G27), UNSW

Sydney NSW 2052, Australia

+61 (2) 9385 0698

m.falster@unsw.edu.au

For peer review only



For peer review only - http://bmjopen.bmj.com/site/about/guidelines.xhtml

# Abstract

**Objective**: Preventable hospitalisations are used internationally as a performance indicator for primary care, but the influence of other health system factors remains poorly understood. This study investigated between-hospital variation in rates of preventable hospitalisation.

**Setting**: Linked health survey and hospital admissions data for a cohort study of 266,826 people aged over 45 years in the state of New South Wales, Australia.

**Method**: Between-hospital variation in preventable hospitalisation was quantified using cross-classified multiple-membership multilevel Poisson models, adjusted for personal sociodemographic, health and area-level contextual characteristics. Variation was also explored for two conditions unlikely to be influenced by discretionary admission practice: emergency admissions for acute myocardial infarction (AMI) and hip fracture.

**Results**: We found significant between-hospital variation in adjusted rates of preventable hospitalisation, with hospitals varying on average 26% from the state mean. Patients served more by community and multipurpose facilities (smaller facilities primarily in rural areas) had higher rates of preventable hospitalisation. Community hospitals had the greatest between-hospital variation, and included the facilities with the highest rates of preventable hospitalisation. There was comparatively little between-hospital variation in rates of admission for AMI and hip fracture.

**Conclusions**: Geographic variation in preventable hospitalisation is determined in part by hospitals, reflecting different roles played by community and multipurpose facilities, compared with major and principal referral hospitals, within the community. Care should be taken when interpreting the indicator simply as a performance measure for primary care.


## Strengths and limitations of the study:

- The use of novel cross-classified multiple membership multilevel models makes this the first study on preventable hospitalisations to have modelled each of patient-, area- and hospital-level effects.
- The use of a large cohort with detailed survey and linked health data allowed adjustment for a large range of patient confounders.
- We had limited data on hospital characteristics and accessibility of primary care
- The study population may not be representative of the Australian population, being an older cohort (age 45 and over) with a low response rate.



For peer review only - http://bmjopen.bmj.com/site/about/guidelines.xhtml

## Introduction

Preventable hospitalisations are an intuitive, yet contentious, performance indicator for primary care. Also known as hospital admissions for ambulatory care sensitive conditions, rates of preventable hospitalisations are used in Australia[1 2] and internationally as a measure of hospital use that could potentially be prevented through timely and effective access to primary care. These admissions are estimated to cost over $30 billion dollars annually in the US,[3] presenting significant potential cost savings to the healthcare system. However, rates of preventable hospitalisation in Australia have not declined, despite accounting for 6% of all hospitalisations and being a national performance indicator for over 10 years.[4]

Health system performance measures should be underpinned by strong evidence that improvements will lead to improvements in health outcomes,[5] and the utility of preventable hospitalisations as a performance measure has been challenged accordingly.[6] Initially developed in the US where large variations in income, workforce and health insurance coverage result in stark disparities in access to primary care,[7 8] the subsequent adoption of the indicator in various international settings has produced a mixed evidence base, particularly in countries with a universal health care system such as Australia[9], Canada[10 11] and the UK.[6] The utility of the indicator is likely to differ according to the characteristics of the patient population, and the barriers and facilitators to accessing care in the health system.

One health system factor which remains poorly understood is the role of hospitals. Differences in a hospital's propensity to admit patients can arise from physician preferences[12] and in-hospital capacity.[7 13 14] Anecdotal reports from the UK suggest that hospitals play a direct role in choosing to admit patients for observation, such as in regional areas where long travel times and limited clinical support can lead to more cautious admission thresholds.[15] Australia has a vast geography, and in remote areas hospitals and emergency departments may be used as a substitute for GP care.[16] However evidence on hospitals' influence on preventable hospitalisations is limited: higher rates have been reported in UK hospitals that convert more emergency department presentations into admissions,[17] and in areas in the US with more hospital beds per capita[18] – although the latter finding has been inconsistent.[19 20]

A better understanding of the role of hospitals would improve our understanding of the limitations of preventable hospitalisations as an indicator of primary care. We sought to quantify between-hospital variation in preventable hospitalisation in New South Wales (NSW), Australia, and assess if this variation differs between categories of hospital facilities.

## Methods

### *Study population*

This observational study included participants in The Sax Institute's 45 and Up Study, a prospective cohort of 267,014 residents of NSW, Australia, aged 45 and over.[21] Eligible participants were randomly selected between 2006-2009 through the Department of Human Services enrolment database. At study entry participants completed a detailed questionnaire containing information on their health and sociodemographic characteristics, and provided informed consent for long-term follow-up, including linkage with administrative health data sets, and use of their data for research purposes.



For peer review only - http://bmjopen.bmj.com/site/about/guidelines.xhtml

For each participant, linked data on hospital admissions (between 2000-2011) and deaths (between 2006-2011) were obtained from the NSW Admitted Patient Data Collection and the NSW Registry of Births Deaths and Marriages mortality data file, respectively. Data linkage was performed probabilistically by the NSW Centre for Health Record Linkage (http://www.cherel.org.au/). Participants were excluded if they had an unknown age, area of residence, or inconsistent records suggesting incorrect linkage (e.g. death before date of study entry).

Ethics approval for the 45 and Up Study was given by the University of New South Wales Human Research Ethics Committee, and ethics approval for this study was given by the NSW Population and Health Services Research Ethics Committee and the University of Western Sydney Research Ethics Committee. All analyses were carried out in accordance with these approvals.

### *Hospitalisations, outcomes and exposures*

Hospital outcomes were identified using the linked hospital admissions data, from the time of participants' entry into the study (between 2006-2009) until death or the end of linked data (31/12/2011), whichever came first. Hospital admissions were restricted to public hospitals only. Transfers and changes in type of care (e.g. from acute to palliative) within a hospital were considered a continuation of the same episode of care.

Preventable hospitalisations were identified according to the 'selected potentially preventable hospitalisations' performance indicator in the Australian National Healthcare Agreement.[22] The indicator is a composite measure of hospital admissions for 21 conditions, including a selection of chronic conditions (e.g. diabetes complications, angina, chronic obstructive pulmonary disease), acute conditions (e.g. dehydration and gastroenteritis, pyelonephritis, cellulitis) and vaccine-preventable conditions (e.g. influenza and pneumonia). Two additional outcome measures, for which hospital admission was unlikely to be influenced by discretionary patterns of care, were used for comparison: emergency admissions for acute myocardial infarction (AMI) and hip fracture.[14] Hospital diagnosis and procedure codes used to identify outcomes are in Appendix 1. Sensitivity analyses tested a recently suggested modification to the preventable hospitalisations indicator, categorising preventable hospitalisations as short (<= 2 days length of stay [LOS]) and long (3+ days LOS), on the basis that shorter admissions may be more amenable to primary prevention.[23]

All person-level information was derived from the self-reported survey completed at study entry, including participants' age, sex, education, marital status, annual household income, employment, language spoken at home, health insurance status, level of social support, body mass index, healthy behaviours, multi-morbidity, functional limitation, self-rated health and psychological distress. These variables reflect patients' predisposition and need to use health services, with most previously found to be associated with preventable hospitalisation.[9] All variables were treated as categorical, with missing values as an additional category.[9]

Area-level information was assigned according to the Statistical Local Area (SLA) of patient residence: geographic remoteness used the Accessibility/Remoteness Index of Australia, and the effective supply of full-time workload equivalent (FWE) general practitioners (GPs). FWE GPs were derived from aggregated Medicare claims data,[9 24] as the number of claims for GP services for residents of each SLA, divided by the average number of claims per FWE GP in NSW. Population estimates were used to calculate the density of FWE GPs per 10,000 residents of each SLA, and divided into quintiles.



For peer review only - http://bmjopen.bmj.com/site/about/guidelines.xhtml

Hospital category was classified according to hospital peer group, a categorisation used for benchmarking and reporting that groups hospitals by the types of services provided.[25] For this analysis, peer groups were collapsed into six broad categories reflecting major differences in the size, role and location of hospitals: principal (>25,000 acute separations per annum), major metropolitan (10-25,000 acute separations per annum), major non-metropolitan (10,000+ acute separations per annum, in rural areas), district (2-10,000 acute separations per annum), community (<2,000 acute separations per annum) and multipurpose (smaller facilities providing integrated acute health, nursing home, hostel, community health, aged care and non-specialised sub-acute services) (detailed definitions in Appendix 2). Australia has a vast geography with most high-volume facilities located in metropolitan and inner regional areas. The smaller community and multipurpose facilities provide a mix of acute and sub-acute care, with multipurpose able to provide a range of integrated care services as negotiated between government, health practitioners and the community.

### *Statistical methods*

Between-hospital variation in admission was analysed using cross-classified multiple membership multilevel Poisson models.[26] All models used number of hospitalisations as the outcome and log of the follow up time as an offset, so as to model 'rates' of admission, and were adjusted for participants' sociodemographic and health characteristics, geographic remoteness and supply of GP services in their area of residence, so the remaining residual variation was that potentially attributable to hospitals.

Multilevel models allow for variation to be partitioned to various 'levels' for analysis, and these models clustered study participants in both their geographic area of residence (SLA) and all potential hospitals of admission. Because a patient could be admitted to any number of hospitals, this clustering was performed using weighted hospital service area networks of all public hospitals servicing the population.[26] Weighting was determined by patterns of patient flow for all-cause admissions at the level of the postal-area.

From these models, hospital-level incidence rate ratios (IRRs) were derived – the admission rate for the hospital relative to the state average rate, taking into account the factors in the model, as well as the size of the hospital's population.[27] The variation between hospital IRRs was measured using the random intercept variance ($\sigma^2$) from the multilevel model, as well as the average relative deviation (ARD) which quantifies, on average, how much these adjusted hospitalisation rates differ from the statewide adjusted admission rate.[28]

Overall IRRs for hospital types were derived by adding parameters for each hospital type in the model. Given the multiple membership structure, the parameters were calculated as the proportion of hospital services provided by each hospital type in the patient's postal-area. Each parameter was centred on the mean group value, and scaled so a single unit increase represents a 10% increase in service provision. All analyses were performed in SAS9.4 and MLwiN v2.35.

### *Patient and public involvement*

Participants in the 45 and Up Study completed a baseline questionnaire and have provided informed consent for the use of their data for research purposes. However, patients and the public were not involved in the design of this study.



For peer review only - http://bmjopen.bmj.com/site/about/guidelines.xhtml

## Results

Of 267,014 participants in the linked dataset, n=119 were excluded because they had unknown area of residence or incompatible dates in the linked data. Participants in 16 postal areas did not have any hospitalizations during follow-up; the 69 participants residing in these areas were excluded, leaving 266,826 for analysis, over an average follow-up of 3.7 years. Mean age, self-reported health and multi-morbidity of study participants were broadly consistent across remoteness categories (Table 1), although participants in remote areas were slightly younger, with poorer health and a higher number of comorbidities. Patients were admitted to a total of 259 different facilities, including n=17 principal referral, n=12 major metropolitan, n=12 major non-metropolitan, n=38 district, n=70 community and n=110 multi-disciplinary facilities.

The majority of the 30,264 preventable hospitalisations during follow-up were to principal hospitals (31%) with only a small proportion to community (9.1%) and multipurpose (2.6%) facilities (Table 1). However, this pattern was inverted for participants in remote and outer regional areas, with the majority of admissions to community (24.6%) and district hospitals (37.4%). A similar pattern was observed in the 3,167 emergency AMI and 1,550 emergency hip fracture admissions, although with a smaller proportion of admissions overall to district, community and multipurpose hospitals (data not shown).

There was significant between-hospital variation in preventable hospitalisation, such that each hospital deviated on average 26% from the mean adjusted rate of admission ($\sigma^2$=0.312; standard error [SE]=0.059; ARD=25.6). This variation was much less pronounced for emergency admissions for AMI ($\sigma^2$=0.047; SE=0.026; ARD=9.6) and was not significant for hip fracture ($\sigma^2$=0.015; SE=0.017; ARD=2.9)

Figure 1 shows hospital-level IRRs from the multilevel model, which indicate how each hospital differs from the state average, after adjusting for patient and geographic factors. There was considerable variation in preventable hospitalisation, with 7% of hospitals having significantly higher or lower than average adjusted rates of admission. When stratified by category of hospital, the greatest variation was seen in community and district hospitals, with community hospitals in particular having the highest rates of preventable hospitalisation – up to 4 times the average rate of admission. There were no hospitals with significant deviations from the mean for emergency AMI or hip fracture admissions.

ARDs stratified by hospital category (Figure 1) corroborated these results, with community hospitals having the highest levels of variation in preventable hospitalisation (average 36% difference from the mean), and principal hospitals varying the least (average 21% difference from the mean). There was less variation between all hospital types for emergency AMI or hip fracture admissions than preventable hospitalisations,.

The inclusion of hospital category in the regression models (Table 2) showed significantly higher rates of preventable hospitalisations among people serviced by community (IRR:1.06; 95% CIs:1.02-1.10) and multipurpose (IRR:1.05; 95% CIs:1.01-1.09) than principal hospitals. For emergency AMI admissions, there were significantly higher rates in people serviced by major non-metropolitan (IRR:1.04; 95% CIs:1.02-1.07), and lower rates among people serviced by multipurpose facilities (IRR:0.93; 95% CIs:0.88-0.99). IRRs for all variables in the model are provided in Appendix 3.



For peer review only - http://bmjopen.bmj.com/site/about/guidelines.xhtml

A sensitivity analysis categorising length of stay (Table 3) found more the majority of preventable hospitalisations (n=16,305, 53.9%) were short stay admissions (0-2 day LOS), with the remainder (n=13,959, 46.1%) having a LOS of three days or more. There were differing patterns of variation by length of stay, with the significantly higher rates of admission for community and multipurpose hospitals restricted to short-stay preventable hospitalisations only.

## Discussion

We found significant variation in rates of preventable hospitalisation between public hospitals, even after adjustment for patient and geographic factors. Our finding was most marked for community and multipurpose hospitals – smaller facilities which provide the majority of services to patients living in regional and remote communities. Given similar variation was not observed for other less-discretionary conditions, major hospitals servicing regional areas, or for admissions with a longer length of stay, our findings indicate a varying propensity to admit patients for preventable hospitalisation among and between categories of hospital facilities.

Our findings do not suggest that preventable hospitalisations should be used as indicator of discretionary admission practice – the effect size was modest and, consistent with prior research, the strongest predictors of admission were patient sociodemographic and health characteristics.[9] But while admissions to community and multipurpose hospitals represented only a small proportion (12%) of all preventable hospitalisations, they made up 55% of admissions in remote areas of Australia, where there is both high variability - with over a five-fold variation in rates of preventable hospitalisations[2] - and also the highest rates of admission.[1 2] Accordingly, these differences in admission practices are likely to play an important role in driving geographic variation in the preventable hospitalisations performance indicator. The implications for performance measurement are clear: interpretation of the indicator is complex and factors along the care continuum, including hospitals' propensity to admit, influence variation in admission rates.

There is very little existing evidence about how admissions for preventable hospitalisations vary between hospitals in Australia. One study of major hospitals in NSW reported up to 11-fold and 7-fold variation between hospitals in the proportion of admissions that were for congestive heart failure and chronic obstructive pulmonary disease respectively,[29] and earlier work from the current team found no association between preventable hospitalisations and hospital bed occupancy rates.[26] Importantly, these previous analyses (as with most hospital reporting) excluded community and multipurpose hospitals - the facilities in this study with the strongest patterns of variation. It is difficult to assess causes of between-hospital variation in the context of this analysis. Both differences in hospital roles (e.g. provision of both acute and sub-acute services) and differences in discretionary admission thresholds (e.g. admitting patients for observation to avoid long travel times[15]) could contribute, as well as the provision of community-based services such as hospital in the home[30].

The preventable hospitalisations indicator is considered a measure of timely and effective access to primary care, and our findings are not inconsistent with this interpretation. Some of the variation in community and multipurpose hospitals is likely to reflect the facility acting as a substitute for primary care in areas where access is poor, and may arguably reflect either a deficiency of primary care or appropriate integration of services to meet population needs. We were unable to examine further dimensions of access, such as waiting times, distance to nearest GP clinic and type of in-



For peer review only - http://bmjopen.bmj.com/site/about/guidelines.xhtml

hospital practitioner, so were unable to further tease out these effects. However our results do suggest that use of the preventable hospitalisations indicator beyond its original intent—as a yardstick measure of health system performance[7]—needs to be approached with caution.

Our study is among a few internationally to provide evidence of a hospital-level difference in propensity to admit patients for preventable hospitalisations,[17 18] and is the first to quantify the extent of this variation. The findings, while not directly applicable to different health care settings, highlight the contextual differences between health systems which should be considered when adopting international performance indicators, as well as the need for localised policy responses tailored to models of care.

The key strength of this study is the use of a large cohort with detailed survey and linked health data. Much inference on preventable hospitalisation is limited either by unmeasured confounders or the use of ecological measures of patient demographics, and estimation of hospital effects can be difficult given the lack of a discrete population denominator. The use of cross-classified multiple membership multilevel models makes this the only study to perform appropriate modelling for each of patient-, area- and hospital-level effects. A limitation is that unexplained hospital variation remained, and we had only limited data on hospital characteristics, so the impact of more complex models of care, such as integrated care programs, has yet to be explored. The use of a population cohort meant further measures of morbidity derived from hospital admissions data (e.g. Charlson index) were not able to be utilised. Generalizability of our findings may also be limited given the older age (45 years and over) and low response rate (18%) of the study cohort, although the considerable size and heterogeneity of the study mean inferences from within-cohort comparisons remain valid.[31]

## Conclusion

Geographic variation in rates of preventable hospitalisation is determined in part by the hospitals themselves, reflecting different roles of smaller and rural hospitals compared with major and principal referral hospitals to meet the needs of the community. International adoption of the preventable hospitalisations health performance indicator should consider the contextual barriers and facilitators to accessing care in the relevant health system. In Australia, care should be taken when interpreting preventable hospitalisations simply as a measure of accessibility and quality of primary care.

For peer review only



For peer review only - http://bmjopen.bmj.com/site/about/guidelines.xhtml

## Funding

This study was funded by a National Health and Medical Research Council Partnership Project Grant (#1036858) and by partner agencies the Australian Commission on Safety and Quality in Health Care, the Agency for Clinical Innovation and the NSW Bureau of Health Information. MF is supported by a National Health and Medical Research Council Early Career Fellowship (#1139133). AL receives funding from the Medical Research Council (MC_UU_12017/13) and the Scottish Government Chief Scientist Office (SPHSU13).

## Acknowledgements

The authors would like to thank Federico Girosi and the Australian Primary Health Care Research Institute at the Australian National University for assistance with the data. This research was completed using data collected through the 45 and Up Study (www.saxinstitute.org.au). The 45 and Up Study is managed by the Sax Institute in collaboration with major partner Cancer Council NSW; and partners: National Heart Foundation of Australia (NSW Division); NSW Ministry of Health; NSW Government Family & Community Services – Ageing, Carers and the Disability Council NSW; and the Australian Red Cross Blood Service. We thank the many thousands of people participating in the 45 and Up Study. We thank the Sax Institute, the NSW Ministry of Health and the NSW Register of Births, Deaths and Marriages for allowing access to the data, and the Centre for Health Record Linkage for conducting the probabilistic linkage of records.

## Data availability

The data set used for this study was constructed from pre-existing source data sets (routinely collected data and the 45 and Up Study) with the permission from the custodians of each of these data sets and with specific ethical approval. The data set could potentially be made available to other researchers if they obtain the necessary approvals. Further information on this process can be obtained from the 45 and Up Study (45andUp.research@saxinstitute.org.au) and the NSW Centre for Health Record Linkage (cherel.mail@moh.health.nsw.gov.au).

## Author contributions

MOF conceived the project, undertook the literature review, performed data analysis and drafted the manuscript. LRJ and AHL provided guidance and interpretation. All three authors edited, reviewed and approved the final manuscript. LRJ conceived the APHID study

## Competing interests

None declared



For peer review only - http://bmjopen.bmj.com/site/about/guidelines.xhtml

# References

1. Australian Commission on Safety and Quality in Health Care and Australian Institute of Health and Welfare. The Second Australian Atlas of Healthcare Variation. Sydney: ACSQHC; 2017.
2. National Health Performance Authority. Healthy Communities: Potentially preventable hospitalisations in 2013–14. Sydney: NHPA; 2015.
3. Torio CM, Elixhauser A, Andrews RM. Trends in potentially preventable hospital admissions among adults and children, 2005–2010. Rockville, MD: Agency for Healthcare Research and Quality, 2013.
4. Australian Institute of Health and Welfare. Admitted patient care 2014–15: Australian hospital statistics. Health services series no. 68. Cat. no. HSE 172. Canberra: AIHW; 2016
5. Baker DW, Chassin MR. Holding Providers Accountable for Health Care Outcomes. *Annals of internal medicine* 2017;167(6):418-23
6. Giuffrida A, Gravelle H, Roland M. Measuring quality of care with routine data: avoiding confusion between performance indicators and health outcomes. *Brit Med J* 1999;319(7202):94-98.
7. Billings J, Zeitel L, Lukomnik J, et al. Impact of socioeconomic status on hospital use in New York City. *Health Affairs (Project Hope)* 1993;12:162-73.
8. Weissman JS, Gatsonis C, Epstein AM. Rates of avoidable hospitalization by insurance status in Massachusetts and Maryland. *JAMA* 1992;268(17):2388-94
9. Falster MO, Jorm LR, Douglas KA, et al. Sociodemographic and health characteristics, rather than primary care supply, are major drivers of geographic variation in preventable hospitalizations in Australia. *Med Care* 2015;53(5):436-45.
10. Roos LL, Walld R, Uhanova J, et al. Physician visits, hospitalizations, and socioeconomic status: ambulatory care sensitive conditions in a canadian setting. *Health Serv Res* 2005;40:1167-85.
11. Trachtenberg AJ, Dik N, Chateau D, et al. Inequities in ambulatory care and the relationship between socioeconomic status and respiratory hospitalizations: a population-based study of a canadian city. *Annals of family medicine* 2014;12(5):402-7.
12. Dean NC, Jones JP, Aronsky D, et al. Hospital admission decision for patients with community-acquired pneumonia: variability among physicians in an emergency department. *Ann Emerg Med* 2012;59(1):35-41.
13. Fisher ES, Wennberg JE, Stukel TA, et al. Associations among hospital capacity, utilization, and mortality of US Medicare beneficiaries, controlling for sociodemographic factors. *Health Serv Res* 2000;34(6):1351-62.
14. Wennberg J, McPherson K, Goodman D. Small Area Analysis and the Challenge of Practice Variation. In: Johnson A, Stukel T, eds. Medical Practice Variations. Boston, MA: Springer US 2016:1-25.
15. Culliton G. Large variance in hospitals' 'propensity to admit'. *Irish Medical Times* 2016.
16. National Health Performance Authority. Healthy Communities: Use of emergency department and GP services in 2013-14. Sydney: NHPA, 2016.
17. O'Cathain A, Knowles E, Maheswaran R, et al. Hospital characteristics affecting potentially avoidable emergency admissions: national ecological study. *Health Services Management Research* 2013;26(4):110-8.
18. Zhan C, Miller MR, Wong H, et al. The effects of HMO penetration on preventable hospitalizations. *Health Serv Res* 2004;39(2):345-61.
19. Basu J, Friedman B, Burstin H. Primary care, HMO enrollment, and hospitalization for ambulatory care sensitive conditions: a new approach. *Med Care* 2002;40(12):1260-9.
20. Berlin C, Busato A, Rosemann T, et al. Avoidable hospitalizations in Switzerland: a small area analysis on regional variation, density of physicians, hospital supply and rurality. *BMC Health Serv Res* 2014;14(1):289.
21. Banks E, Redman S, Jorm L, et al. Cohort profile: the 45 and up study. *International Journal of Epidemiology* 2008;37(5):941-7.

For peer review only

22. Australian Institute of Health and Welfare. National Healthcare Agreement: PI 22-Selected potentially preventable hospitalisations, 2012 2012 [Available from: http://meteor.aihw.gov.au/content/index.phtml/itemId/443687 accessed 18/05/2012 2012.
23. Swerissen H, Duckett S, Wright J. Chronic failure in primary care: Grattan Institute, 2016.
24. Mazumdar S, Konings P, Butler D, et al. General practitioner (family physician) workforce in Australia: comparing geographic data from surveys, a mailing list and medicare. *BMC Health Serv Res* 2013;13(1):343.
25. NSW Health. NSW Health Services Comparison Data Book 2008/2009. North Sydney, NSW: Demand and Performance Evaluation, NSW Health, 2010.
26. Falster MO, Jorm LR, Leyland AH. Using Weighted Hospital Service Area Networks to Explore Variation in Preventable Hospitalization. *Health Serv Res* 2018;53 Suppl 1:3148-69.
27. Merlo J, Chaix B, Yang M, et al. A brief conceptual tutorial of multilevel analysis in social epidemiology: linking the statistical concept of clustering to the idea of contextual phenomenon. *J Epidemiol Community Health* 2005;59(6):443-9.
28. Tarkiainen L, Martikainen P, Laaksonen M, et al. Comparing the effects of neighbourhood characteristics on all-cause mortality using two hierarchical areal units in the capital region of Helsinki. *Health Place* 2010;16(2):409-12.
29. Bureau for Health Information. Chronic disease care: A piece of the picture. Sydney: BHI, 2011.
30. NSW Ministry of Health. Hospital in the Home Guideline. North Sydney: NSW Ministry of Health, 2013.
31. Mealing NM, Banks E, Jorm LR, et al. Investigation of relative risk estimates from studies of the same population with contrasting response rates and designs. *BMC Medical Research Methodology* 2010;10:26.

For peer review only



For peer review only - http://bmjopen.bmj.com/site/about/guidelines.xhtml

# Tables and Figures

***Table 1: Cohort characteristics at baseline, and number of preventable hospitalisations during follow-up, by remoteness of area of residence***

| | Total | By remoteness category of residence | | | |
|---|---|---|---|---|---|
| | | Major cities | Inner regional | Outer regional | Remote |
| **Cohort characteristics** | | | | | |
| N | 266,826 | 119,496 | 94,568 | 47,438 | 5,324 |
| Age (mean) | 62.7 | 63.4 | 62.4 | 62.2 | 60.7 |
| Age (IQR) | 53.6-70.4 | 53.6-71.9 | 53.8-69.7 | 53.7-69.4 | 52.0-67.8 |
| % Female | 53.6 | 52.4 | 54.7 | 54.3 | 55.5 |
| % fair/poor self-rated health | 13.7 | 13.9 | 13.4 | 13.7 | 16.1 |
| % with >3 comorbidities | 7.4 | 7.3 | 7.5 | 7.2 | 8.0 |
| **Preventable hospitalisations** | | | | | |
| Number of admissions | 30,264 | 12,512 | 10,161 | 6,512 | 1,079 |
| Admissions to hospital type (%) | | | | | |
| - Principal | 9,398 (31.0) | 7,506 (60.0) | 1,600 (15.7) | 255 (3.9) | 37 (3.4) |
| - Major metropolitan | 4,172 (13.8) | 3,321 (26.5) | 787 (7.7) | 61 (0.9) | 3 (0.3) |
| - Major non-metropolitan | 6,443 (21.3) | 560 (4.5) | 3,933 (38.7) | 1,872 (28.7) | 78 (7.2) |
| - District | 6,715 (22.2) | 804 (6.4) | 3,070 (30.2) | 2,468 (37.9) | 373 (34.6) |
| - Community | 2,760 (9.1) | 278 (2.2) | 611 (6.1) | 1,491 (22.9) | 380 (35.2) |
| - Multipurpose | 776 (2.6) | 43 (0.3) | 160 (1.6) | 365 (5.6) | 208 (19.3) |

For peer review only

*Table 2: Incidence rate ratio (IRR) of hospital category for preventable hospitalisation and emergency admissions for acute myocardial infarction (AMI) and hip fracture*

| | Preventable hospitalisations | | AMI (emergency) | | Hip fracture (emergency) | |
|---|---|---|---|---|---|---|
| | IRR | (95% CIs) | IRR | (95% CIs) | IRR | (95% CIs) |
| Hospital category | | | | | | |
| Principal | 1.00 | (ref) | 1.00 | (ref) | 1.00 | (ref) |
| Major metropolitan | 0.99 | (0.95 – 1.03) | 1.02 | (0.99 – 1.05) | 1.02 | (0.99 – 1.05) |
| Major non-metropolitan | 1.01 | (0.97 – 1.04) | 1.04 | (1.02 – 1.07) | 0.99 | (0.96 – 1.02) |
| District | 1.02 | (0.99 – 1.06) | 1.00 | (0.97 – 1.03) | 0.99 | (0.96 – 1.02) |
| Community | 1.06 | (1.02 – 1.10) | 0.97 | (0.93 – 1.01) | 0.96 | (0.91 – 1.01) |
| Multipurpose | 1.05 | (1.01 – 1.09) | 0.93 | (0.88 – 0.99) | 1.02 | (0.94 – 1.09) |

For peer review only



For peer review only - http://bmjopen.bmj.com/site/about/guidelines.xhtml

*Table 3: Average relative deviation (ARD) and Incidence rate ratio (IRR) by hospital category for rates of preventable hospitalisation, separated as short-stay (0-2 days length of stay) and long-stay (>2 days length of stay) admissions*

| | Short stay (0-2 days length of stay) | | | Long stay (>2 days length of stay) | | |
|---|---|---|---|---|---|---|
| | ARD | IRR | (95% CIs) | ARD | IRR | (95% CIs) |
| Hospital category | | | | | | |
| Principal | 17.9 | 1.00 | (ref) | 14.6 | 1.00 | (ref) |
| Major metropolitan | 25.5 | 0.99 | (0.95 – 1.02) | 25.9 | 1.00 | (0.97 – 1.03) |
| Major non-metropolitan | 22.7 | 1.02 | (0.98 – 1.05) | 11.3 | 0.99 | (0.96 – 1.02) |
| District | 30.4 | 1.02 | (0.99 – 1.05) | 24.3 | 0.98 | (0.95 – 1.00) |
| Community | 17.5 | 1.04 | (1.01 – 1.07) | 25.7 | 1.02 | (0.99 – 1.05) |
| Multipurpose | 24.3 | 1.04 | (1.00 – 1.08) | 11.6 | 0.99 | (0.95– 1.03) |

For peer review only

***Figure 1: Hospital-specific incidence rate ratios from the mean adjusted rate of admission, for preventable hospitalisation and emergency admissions for acute myocardial infarction (AMI) and hip fracture, overall and stratified by hospital category***

ARD = average relative deviation. Red and blue markers indicate hospitals with significantly higher and lower rates of admission respectively. Adjusted for patient sociodemographic and health factors, remoteness and supply of GP services in area of residence

For peer review only


For peer review only - http://bmjopen.bmj.com/site/about/guidelines.xhtml

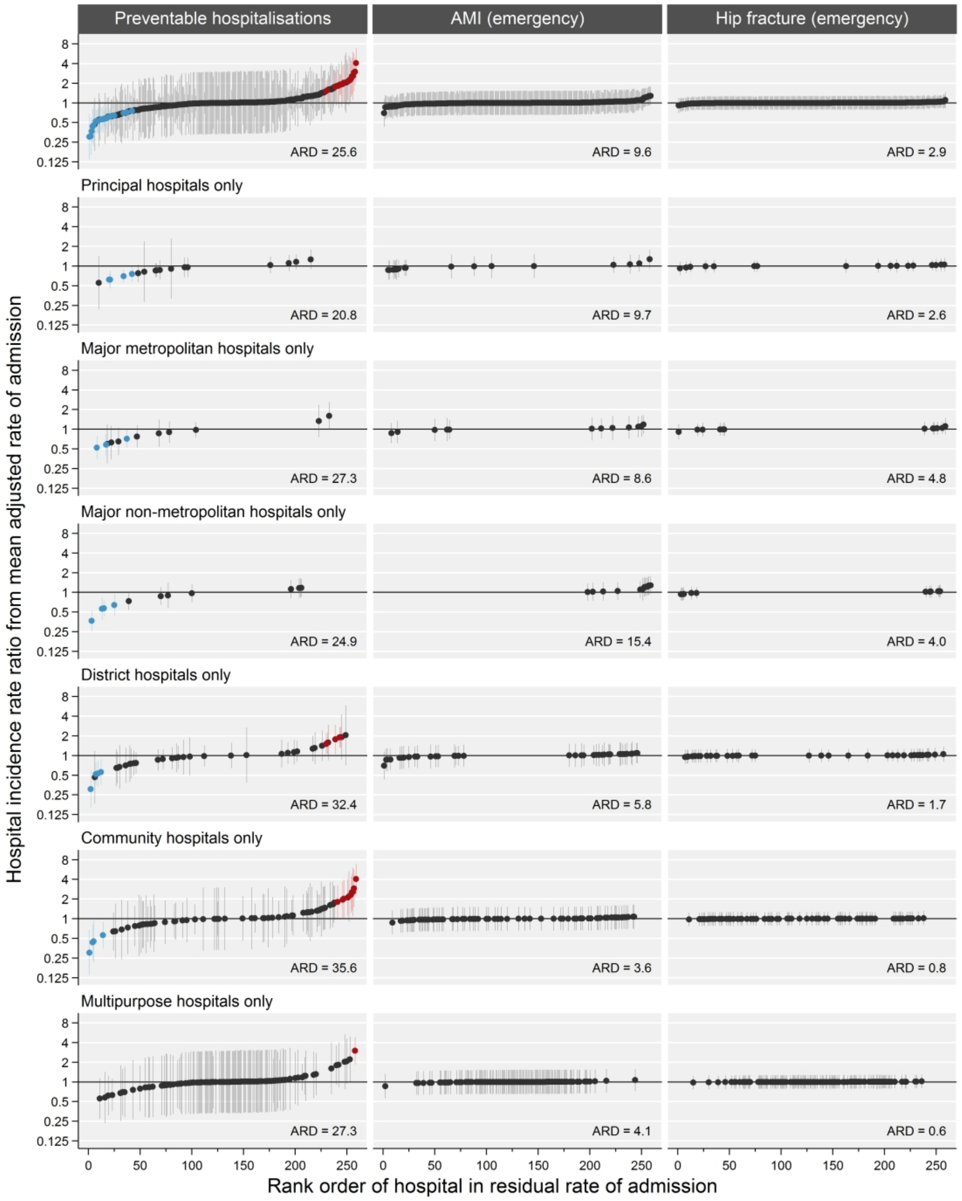


Hospital-specific incidence rate ratios from the mean adjusted rate of admission, for preventable hospitalisation and emergency admissions for acute myocardial infarction (AMI) and hip fracture, overall and stratified by hospital type

330x412mm (300 x 300 DPI)

# Online appendices

## Appendix 1:

ICD-10-AM codes for identifying hospital outcomes.

| Category | ICD-10-AM diagnosis and procedure codes |
|---|---|
| **Preventable hospitalisations** | |
| Angina | I20, I24.0, I24.8, I24.9 as principal diagnosis only, exclude cases with procedure codes not in blocks [1820] to [2016] |
| Asthma | J45, J46 as principal diagnosis only |
| Chronic obstructive pulmonary disease (COPD) | J20, J41, J42, J43, J44, J47 as principal diagnosis only, J20 only with additional diagnoses of J41, J42, J43,J44, J47 |
| Congestive cardiac failure | I50, I11.0, J81 as principal diagnosis only, exclude cases with the following procedure codes: 33172-00, 35304-00, 35305-00, 35310-02, 35310-00, 38281-11, 38281-07, 38278-01, 38278-00, 38281-02, 38281-01, 38281-00, 38256-00, 38278-03, 38284-00, 38284-02, 38521-09, 38270-01, 38456-19, 38456-15, 38456-12, 38456-11, 38456-10, 38456-07, 38456-01, 38470-00, 38475-00, 38480-02, 38480-01, 38480-00, 38488-06, 38488-04, 38489-04, 38488-02, 38489-03, 38487-00, 38489-02, 38488-00, 38489-00, 38490-00, 38493-00, 38497-04, 38497-03, 38497-02, 38497-01, 38497-00, 38500-00, 38503-00, 38505-00, 38521-04, 38606-00, 38612-00, 38615-00, 38653-00, 38700-02, 38700-00, 38739-00, 38742-02, 38742-00, 38745-00, 38751-02, 38751-00, 38757-02, 38757-01, 38757-00, 90204-00, 90205-00, 90219-00, 90224-00, 90214-00, 90214-02. |
| Diabetes complications | E10–E14.9 as principal diagnoses, and E10–E14.9 as additional diagnoses where the principal diagnosis was: hypersmolarity (E87.0), acidosis (E87.2), transient ischaemic attack (G45), nerve disorders and neuropathies (G50–G64), cataracts and lens disorders (H25–H28), retinal disorders (H30–H36), glaucoma (H40–H42), myocardial infarction (I21–I22), other coronary heart diseases (I20, I23–I25), heart failure (I50), stroke and sequelae (I60–I64, I69.0–I69.4), peripheral vascular disease (I70–I74), gingivitis and periodontal disease (K05), kidney diseases including end-stage renal disease (N00–N29), and renal dialysis (Z49) |
| Hypertension | I10, I11.9 as principal diagnosis only, exclude cases with procedure codes according to the list of procedures excluded from the Congestive cardiac failure category above. |
| Iron deficiency anaemia | D50.1, D50.8, D50.9 as principal diagnosis only. |
| Nutritional deficiencies | E40, E41, E42, E43, E55.0, E64.3 as principal diagnosis only. |
| Rheumatic heart disease | I00 to I09 as principal diagnosis only. (Note: includes acute rheumatic fever) |
| Appendicitis with generalised peritonitis | K35.0 in any diagnosis field |
| Cellulitis | L03, L04, L08, L88, L98.0, L98.3 as principal diagnosis only, exclude cases with any procedure except those in blocks 1820 to 2016 or if procedure is 30216-02, 30676-00, 30223-02, 30064-00, 34527-01, 34527-00, 90661-00 and this is the only listed procedure |
| Convulsions and epilepsy | G40, G41, O15, R56 as principal diagnosis only |
| Dehydration and gastroenteritis | A09.9, E86, K52.2, K52.8, K52.9 as principal diagnosis only. |
| Dental conditions | K02, K03, K04, K05, K06, K08, K09.8, K09.9, K12, K13 as principal diagnosis only. |
| Ear, nose and throat infections | H66, H67, J02, J03, J06, J31.2 as principal diagnosis only. |
| Gangrene | R02 in any diagnosis field |
| Pelvic inflammatory disease | N70, N73, N74 as principal diagnosis only. |
| Perforated/bleeding ulcer | K25.0, K25.1, K25.2, K25.4, K25.5, K25.6, K26.0, K26.1, K26.2, K26.4, K26.5, K26.6, K27.0, K27.1, K27.2, K27.4, K27.5, K27.6, K28.0, K28.1, K28.2, K28.4, K28.5, K28.6 as principal diagnosis only. |
| Pyelonephritis | N10, N11, N12, N13.6, N39.0 as principal diagnosis only. |
| Influenza and pneumonia | J10, J11, J13, J14, J15.3, J15.4, J15.7, J15.9, J16.8, J18.1, J18.8 in any diagnosis field, excludes cases with additional diagnosis of D57 (sickle-cell disorders) and people under 2 months |
| Other vaccine-preventable conditions | A35, A36, A37, A80, B05, B06, B16.1, B16.9, B18.0, B18.1, B26, G00.0, M01.4 in any diagnosis field |
| Acute myocardial infarction (AMI) | I21 |
| Hip fracture | S72.0, S72.1, S72.2 |



For peer review only - http://bmjopen.bmj.com/site/about/guidelines.xhtml

# Appendix 2:

Hospital categories, corresponding peer groups from the *NSW Health Services Comparison Data Book 2008/2009*, and number all cause admissions during follow-up.

| Hospital Peer Group | Description | Admissions |
|---|---|---|
| **Principal** | | |
| A1a Principal Referral Group A | Acute hospitals, treating 25,000 or more acute casemix weighted separations per annum, with an average cost weight greater than 1 and having more than 1 specialty service. | 76,193 |
| A1b Principal Referral Group B | Acute hospitals, treating 25,000 or more acute casemix weighted separations per annum, with an average cost weight greater than 1 and 1 or fewer specialty services. | 28,424 |
| A2 Paediatric Specialist | Establishments where the primary role is to provide specialist acute care services for children. | - |
| A3 Ungrouped Acute | Establishments whose primary role is the provision of acute services of a specialised nature for which there is insufficient peers to form additional peer groups. | 6,284 |
| **Major metropolitan** | | |
| B1 Major Metropolitan: | Acute hospitals, treating 10,000 or more acute casemix weighted separations per annum, but having less than 25,000 acute casemix weighted separations or an average casemix weight of less than 1. | 30,512 |
| **Major non-metropolitan** | | |
| B2 Major Non-Metropolitan: | Acute hospitals treating 10,000 or more acute casemix weighted separations per annum that are located in rural areas providing acute specialist and referral services for a catchment population from a large geographical area. | 68,460 |
| **District** | | |
| C1 District Group 1 | Acute hospitals, treating 5,000 or more, but less than 10,000 acute casemix weighted separations per annum. | 27,671 |
| C2 District Group 2 | Acute hospitals, treating 2,000 or more, but less than 5,000 acute casemix weighted separations per annum, plus acute hospitals treating less than 2,000 acute casemix weighted separations per annum but with more than 2,000 separations per annum. | 29,470 |
| **Community** | | |
| D1a Community Acute with Surgery | Acute hospitals, treating less than 2,000 acute casemix weighted separations per annum, and less than 2,000 acute separations per annum, with less than 40% nonacute and outlier bed days of total bed days and greater than 2% of their acute weighted separations being surgical. | 6,352 |
| D1b Community Acute without Surgery | Acute hospitals, treating less than 2,000 acute casemix weighted separations per annum, and less than 2,000 acute separations per annum, with less than 40% nonacute and outlier bed days of total bed days, and less than 2% of their acute weighted separations being surgical. | 3,620 |
| D2 Community Non-Acute | Non-acute hospitals, treating less than 2,000 acute casemix weighted separations per annum, and less than 2,000 acute separations per annum, with more than 40% nonacute and outlier bed days of total bed days. | 3,061 |
| **Multi-purpose** | | |
| F1 Psychiatric | Establishments devoted primarily to the treatment and care of inpatients with psychiatric, mental or behavioural disorders. Centres of non-acute treatment of drug dependence, developmental and intellectual disability are not included here. This group also excludes institutions mainly providing living quarters or day care. | 195 |
| F2 Nursing Homes | Establishments which provide long-term care involving regular base nursing care to chronically ill, frail, disabled or convalescent persons or senile inpatients. They must be approved by the Commonwealth Department of Health and Family Services and /or licensed by the State, or controlled by government departments. | 184 |
| F3 Multi-Purpose Services | Multi-Purpose Services (MPSs) which provide integrated acute health, nursing home, hostel, community health and aged care services under one organisational structure, as agreed between the Commonwealth and State Governments. MPSs provide a range of services which are negotiated with the community, the service providers and the relevant Departments. | 4,493 |
| F4 Sub Acute | Establishments that primarily provide sub-acute services, but are not specialist palliative care or specialist rehabilitation establishments. | 2,588 |
| F5 Palliative Care | Establishments with a specific function of providing palliative care to terminally ill patients. | 86 |
| F6 Rehabilitation | Establishments with a primary role in providing services to persons with an impairment, disability or handicap where the primary goal is improvement in functional status. | 34 |
| F7 Mothercraft | Establishments where the primary role is to help mothers acquire mothercraft skills in an inpatient setting. | 5 |
| F8 Ungrouped Non-Acute | Establishments whose primary role is the provision of non-acute services, but for which there are insufficient peers to form an addition peer group. Limited comparisons can be made within this peer group and with other non-acute facilities. | 328 |



For peer review only - http://bmjopen.bmj.com/site/about/guidelines.xhtml

# Appendix 3:

Incidence rate ratio (IRR) of patient-, area- and hospital-level factors from cross-classified multiple membership multilevel Poisson models on preventable hospitalisations and emergency admissions for acute myocardial infarction (AMI) and hip fracture

| | Preventable hospitalisation | | AMI (emergency) | | Hip fracture (emergency) | |
|---|---|---|---|---|---|---|
| | IRR | (95% CIs) | IRR | (95% CIs) | IRR | (95% CIs) |
| **Patient-level factors** | | | | | | |
| Age | | | | | | |
| 45-54 years | 1.00 | (ref) | 1.00 | (ref) | 1.00 | (ref) |
| 55-64 years | 1.23 | (1.18 - 1.29) | 1.71 | (1.49 - 1.97) | 2.25 | (1.52 - 3.33) |
| 65-74 years | 1.72 | (1.64 - 1.81) | 2.39 | (2.05 - 2.80) | 7.12 | (4.89 - 10.4) |
| 75-84 years | 2.57 | (2.44 - 2.70) | 4.23 | (3.61 - 4.96) | 24.7 | (17.1 - 35.7) |
| 85 years and over | 3.40 | (3.20 - 3.61) | 7.64 | (6.35 - 9.18) | 49.2 | (33.7 - 71.6) |
| Sex | | | | | | |
| Males | 1.00 | (ref) | 1.00 | (ref) | 1.00 | (ref) |
| Females | 0.72 | (0.70 - 0.74) | 0.42 | (0.39 - 0.46) | 1.36 | (1.21 - 1.53) |
| Highest level of education | | | | | | |
| Did not complete high school | 1.00 | (ref) | 1.00 | (ref) | 1.00 | (ref) |
| High school or equivalent | 0.93 | (0.91 - 0.96) | 0.92 | (0.85 - 0.99) | 1.02 | (0.91 - 1.15) |
| University or higher | 0.86 | (0.83 - 0.90) | 0.78 | (0.69 - 0.88) | 1.16 | (0.98 - 1.38) |
| Unknown / missing | 1.13 | (1.07 - 1.20) | 0.95 | (0.77 - 1.16) | 0.90 | (0.68 - 1.18) |
| Marital status | | | | | | |
| Single | 1.00 | (ref) | 1.00 | (ref) | 1.00 | (ref) |
| Married / partnered | 1.19 | (1.13 - 1.25) | 1.09 | (0.93 - 1.28) | 1.31 | (1.05 - 1.63) |
| Widowed / divorced / separated | 1.17 | (1.13 - 1.20) | 1.19 | (1.10 - 1.30) | 1.14 | (1.01 - 1.28) |
| Unknown / missing | 1.22 | (1.08 - 1.37) | 0.79 | (0.50 - 1.25) | 0.57 | (0.25 - 1.29) |
| Annual household income | | | | | | |
| <$10,000 | 1.00 | (ref) | 1.00 | (ref) | 1.00 | (ref) |
| $10,000 - $29,999 | 0.89 | (0.86 - 0.92) | 0.87 | (0.76 - 0.99) | 1.04 | (0.87 - 1.25) |
| $30,000 - $49,000 | 0.80 | (0.76 - 0.84) | 0.89 | (0.76 - 1.04) | 0.80 | (0.62 - 1.02) |
| $50,000 - $69,999 | 0.75 | (0.70 - 0.80) | 0.85 | (0.70 - 1.03) | 0.88 | (0.63 - 1.21) |
| $70,000 or more | 0.65 | (0.61 - 0.69) | 0.75 | (0.62 - 0.90) | 0.85 | (0.63 - 1.16) |
| Not specified | 0.94 | (0.90 - 0.98) | 1.04 | (0.90 - 1.20) | 1.05 | (0.86 - 1.28) |
| Unknown / missing | 1.13 | (1.07 - 1.18) | 1.14 | (0.97 - 1.34) | 1.15 | (0.93 - 1.43) |
| Employment status | | | | | | |
| Not working | 1.00 | (ref) | 1.00 | (ref) | 1.00 | (ref) |
| Part time | 0.82 | (0.79 - 0.86) | 0.90 | (0.78 - 1.04) | 0.63 | (0.47 - 0.84) |
| Full time | 0.84 | (0.80 - 0.88) | 1.16 | (1.01 - 1.33) | 0.90 | (0.67 - 1.19) |
| Missing / unknown | 0.93 | (0.85 - 1.01) | 1.03 | (0.78 - 1.38) | 0.91 | (0.64 - 1.28) |
| Language spoken at home | | | | | | |
| English | 1.00 | (ref) | 1.00 | (ref) | 1.00 | (ref) |
| Other | 0.92 | (0.88 - 0.96) | 0.99 | (0.87 - 1.12) | 1.04 | (0.88 - 1.23) |
| Health insurance status | | | | | | |
| Private with extras | 1.00 | (ref) | 1.00 | (ref) | 1.00 | (ref) |
| Private, no extras | 1.03 | (0.99 - 1.08) | 1.05 | (0.93 - 1.18) | 0.93 | (0.79 - 1.09) |
| Department of Veterans Affairs | 1.28 | (1.21 - 1.36) | 1.08 | (0.91 - 1.28) | 1.11 | (0.91 - 1.35) |
| Health Care Card | 1.60 | (1.54 - 1.65) | 1.42 | (1.29 - 1.57) | 0.94 | (0.82 - 1.08) |



For peer review only - http://bmjopen.bmj.com/site/about/guidelines.xhtml

For peer review only

| | Preventable hospitalisation | | AMI (emergency) | | Hip fracture (emergency) | |
|---|---|---|---|---|---|---|
| | IRR | (95% CIs) | IRR | (95% CIs) | IRR | (95% CIs) |
| None | 1.50 | (1.44 - 1.55) | 1.26 | (1.13 - 1.41) | 0.93 | (0.78 - 1.10) |
| Number of people can depend on | | | | | | |
| 0 people | 1.00 | (ref) | 1.00 | (ref) | 1.00 | (ref) |
| 1-4 people | 1.09 | (1.04 - 1.14) | 0.92 | (0.80 - 1.06) | 1.20 | (0.96 - 1.49) |
| 5-10 people | 1.07 | (1.02 - 1.12) | 0.92 | (0.80 - 1.07) | 1.18 | (0.94 - 1.47) |
| 11 or more people | 1.20 | (1.14 - 1.27) | 0.89 | (0.75 - 1.06) | 1.15 | (0.89 - 1.50) |
| Unknown / missing | 1.16 | (1.10 - 1.24) | 0.99 | (0.82 - 1.20) | 1.35 | (1.04 - 1.77) |
| Positive health seeking behaviours [a] | | | | | | |
| 0 health behaviours | 0.96 | (0.86 - 1.07) | 1.02 | (0.72 - 1.43) | 1.31 | (0.77 - 2.23) |
| 1 health behaviour | 1.00 | (ref) | 1.00 | (ref) | 1.00 | (ref) |
| 2 health behaviours | 0.89 | (0.86 - 0.93) | 0.89 | (0.80 - 1.00) | 0.76 | (0.65 - 0.89) |
| 3 health behaviours | 0.78 | (0.75 - 0.81) | 0.76 | (0.68 - 0.85) | 0.65 | (0.55 - 0.77) |
| 4 health behaviours | 0.76 | (0.72 - 0.80) | 0.75 | (0.64 - 0.88) | 0.55 | (0.43 - 0.70) |
| BMI | | | | | | |
| Underweight | 1.13 | (1.08 - 1.17) | 1.01 | (0.88 - 1.15) | 1.10 | (0.96 - 1.27) |
| Healthy weight | 1.00 | (ref) | 1.00 | (ref) | 1.00 | (ref) |
| Overweight | 0.94 | (0.91 - 0.97) | 0.95 | (0.87 - 1.03) | 0.57 | (0.50 - 0.64) |
| Obese | 1.00 | (0.96 - 1.03) | 1.00 | (0.90 - 1.10) | 0.35 | (0.29 - 0.42) |
| Unknown / missing | 1.20 | (1.10 - 1.32) | 1.10 | (0.80 - 1.50) | 0.69 | (0.41 - 1.16) |
| Self-rated health | | | | | | |
| Excellent | 1.00 | (ref) | 1.00 | (ref) | 1.00 | (ref) |
| Very good | 1.22 | (1.13 - 1.30) | 1.21 | (1.03 - 1.43) | 0.86 | (0.67 - 1.10) |
| Good | 1.60 | (1.50 - 1.71) | 1.32 | (1.12 - 1.55) | 1.04 | (0.81 - 1.33) |
| Fair | 2.58 | (2.40 - 2.76) | 1.59 | (1.33 - 1.90) | 1.18 | (0.91 - 1.54) |
| Poor | 4.10 | (3.79 - 4.43) | 1.84 | (1.47 - 2.32) | 1.66 | (1.20 - 2.29) |
| Unknown / missing | 2.27 | (2.09 - 2.46) | 1.37 | (1.09 - 1.72) | 1.04 | (0.77 - 1.42) |
| Multi-morbid conditions [b] | | | | | | |
| No conditions | 1.00 | (ref) | 1.00 | (ref) | 1.00 | (ref) |
| 1 condition | 1.35 | (1.31 - 1.40) | 1.20 | (1.09 - 1.32) | 1.00 | (0.87 - 1.14) |
| 2 conditions | 1.98 | (1.91 - 2.05) | 1.45 | (1.30 - 1.62) | 0.90 | (0.77 - 1.05) |
| 3 or more conditions | 2.71 | (2.60 - 2.82) | 2.26 | (2.01 - 2.55) | 1.06 | (0.90 - 1.26) |
| Functional limitations [c] | | | | | | |
| No limitation | 1.00 | (ref) | 1.00 | (ref) | 1.00 | (ref) |
| Minor limitations | 1.04 | (0.97 - 1.10) | 0.97 | (0.84 - 1.14) | 0.83 | (0.60 - 1.16) |
| Mild limitation | 1.25 | (1.18 - 1.32) | 1.04 | (0.91 - 1.19) | 1.13 | (0.86 - 1.48) |
| Moderate limitation | 1.56 | (1.48 - 1.64) | 1.17 | (1.03 - 1.34) | 1.86 | (1.45 - 2.38) |
| Severe limitation | 2.36 | (2.24 - 2.48) | 1.49 | (1.30 - 1.71) | 2.83 | (2.21 - 3.63) |
| Unknown / missing | 1.71 | (1.61 - 1.80) | 1.24 | (1.07 - 1.44) | 1.75 | (1.36 - 2.26) |
| Psychological distress [d] | | | | | | |
| Low distress | 1.00 | (ref) | 1.00 | (ref) | 1.00 | (ref) |
| Moderate distress | 1.03 | (0.99 - 1.06) | 1.01 | (0.91 - 1.12) | 1.03 | (0.88 - 1.20) |
| High distress | 0.99 | (0.95 - 1.03) | 1.08 | (0.92 - 1.26) | 1.10 | (0.87 - 1.39) |
| Very high distress | 0.99 | (0.94 - 1.06) | 0.88 | (0.69 - 1.12) | 1.18 | (0.83 - 1.66) |
| Unknown / missing | 1.09 | (1.03 - 1.15) | 1.31 | (1.12 - 1.54) | 1.16 | (0.95 - 1.42) |
| **Area-level factors** | | | | | | |
| Remoteness of residence | | | | | | |


For peer review only - http://bmjopen.bmj.com/site/about/guidelines.xhtml
For peer review only

| | Preventable hospitalisation | | AMI (emergency) | | Hip fracture (emergency) | |
|---|---|---|---|---|---|---|
| | IRR | (95% CIs) | IRR | (95% CIs) | IRR | (95% CIs) |
| Major city | 1.00 | (ref) | 1.00 | (ref) | 1.00 | (ref) |
| Inner regional | 0.95 | (0.88 - 1.02) | 1.05 | (0.91 - 1.22) | 1.13 | (0.95 - 1.33) |
| Outer regional | 1.01 | (0.88 - 1.15) | 1.08 | (0.87 - 1.34) | 1.06 | (0.81 - 1.38) |
| Remote / very remote | 1.24 | (0.97 - 1.58) | 0.95 | (0.61 - 1.48) | 0.84 | (0.44 - 1.62) |
| Full-time workload equivalent GPs | | | | | | |
| Quintile 1 (2.64-6.90 GPs) | 1.00 | (ref) | 1.00 | (ref) | 1.00 | (ref) |
| Quintile 2 (6.91-7.60 GPs) | 0.89 | (0.78 - 1.02) | 0.88 | (0.73 - 1.07) | 1.01 | (0.80 - 1.29) |
| Quintile 3 (7.63-8.64 GPs) | 0.95 | (0.84 - 1.08) | 0.89 | (0.74 - 1.08) | 1.10 | (0.87 - 1.39) |
| Quintile 4 (8.65-9.94 GPs) | 0.92 | (0.79 - 1.06) | 0.88 | (0.72 - 1.07) | 1.04 | (0.82 - 1.32) |
| Quintile 5 (9.95-13.3 GPs) | 1.02 | (0.87 - 1.19) | 0.91 | (0.74 - 1.12) | 0.96 | (0.74 - 1.23) |
| **Hospital-level factors** | | | | | | |
| Hospital category | | | | | | |
| Principal | 1.00 | (ref) | 1.00 | (ref) | 1.00 | (ref) |
| Major metropolitan | 0.99 | (0.95 - 1.03) | 1.02 | (0.99 - 1.05) | 1.02 | (0.99 - 1.05) |
| Major non-metropolitan | 1.01 | (0.97 - 1.04) | 1.04 | (1.02 - 1.07) | 0.99 | (0.96 - 1.02) |
| District | 1.02 | (0.99 - 1.06) | 1.00 | (0.97 - 1.03) | 0.99 | (0.96 - 1.02) |
| Community | 1.06 | (1.02 - 1.10) | 0.97 | (0.93 - 1.01) | 0.96 | (0.91 - 1.01) |
| Multipurpose | 1.05 | (1.01 - 1.09) | 0.93 | (0.88 - 0.99) | 1.02 | (0.94 - 1.09) |
| **Random effects** | | | | | | |
| Residual random effect (SE) | | | | | | |
| Hospital-level | 0.276 | (0.056) | 0.010 | (0.013) | 0.024 | (0.022) |
| Area-level | 0.061 | (0.011) | 0.050 | (0.015) | 0.013 | (0.011) |

[a] Healthy behaviours, of non-smoking status, safe level of alcohol consumption (<14 drinks per week), at least 2.5 hours of intensity-weighted physical activity per week, and meeting dietary guidelines for daily fruit (2 serves) and vegetable (5 serves) consumption

[b] Of self-reported heart disease, high blood pressure, stroke, diabetes, blood clot, asthma, Parkinson's disease, and any cancer except skin cancer.

[c] Measured using the Medical Outcome Study physical functioning scale.

[d] Measured using the K10 scale.



For peer review only - http://bmjopen.bmj.com/site/about/guidelines.xhtml
